# Poly(styrene)-*block*-Maltoheptaose Films for Sub-10 nm Pattern Transfer: Implications for Transistor Fabrication

**DOI:** 10.1021/acsanm.1c00582

**Published:** 2021-05-13

**Authors:** Anette Löfstrand, Reza Jafari Jam, Karolina Mothander, Tommy Nylander, Muhammad Mumtaz, Alexei Vorobiev, Wen-Chang Chen, Redouane Borsali, Ivan Maximov

**Affiliations:** † NanoLund and Solid State Physics, Lund University, SE-221 00 Lund, Sweden; ‡ NanoLund and Physical Chemistry, Lund University, P.O. Box 124, SE-221 00 Lund, Sweden; § Univ. Grenoble Alpes, CNRS, CERMAV, 38000 Grenoble, France; ∥ Division for Materials Physics, Department of Physics and Astronomy, Uppsala University, P.O. Box 516, SE-751 20 Uppsala, Sweden; ⊥ Advanced Research Center for Green Materials Science and Technology, National Taiwan University, Taipei 10617, Taiwan

**Keywords:** block copolymer lithography, sequential infiltration synthesis, neutron reflectometry, carbohydrate, maltoheptaose, reactive ion etching, sub-10 nm pattern transfer

## Abstract

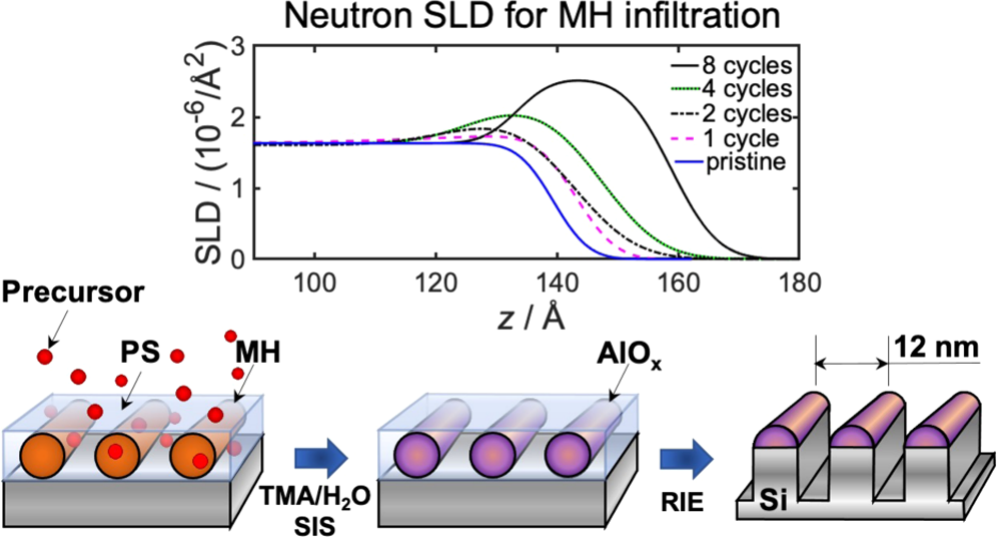

Sequential infiltration synthesis (SIS) into poly(styrene)-*block*-maltoheptaose (PS-*b*-MH) block copolymer using vapors of trimethyl aluminum and water was used to prepare nanostructured surface layers. Prior to the infiltration, the PS-*b*-MH had been self-assembled into 12 nm pattern periodicity. Scanning electron microscopy indicated that horizontal alumina-like cylinders of 4.9 nm diameter were formed after eight infiltration cycles, while vertical cylinders were 1.3 nm larger. Using homopolymer hydroxyl-terminated poly(styrene) (PS–OH) and MH films, specular neutron reflectometry revealed a preferential reaction of precursors in the MH compared to PS–OH. The infiltration depth into the maltoheptaose homopolymer film was found to be 2.0 nm after the first couple of cycles. It reached 2.5 nm after eight infiltration cycles, and the alumina incorporation within this infiltrated layer corresponded to 23 vol % Al_2_O_3_. The alumina-like material, resulting from PS-*b*-MH infiltration, was used as an etch mask to transfer the sub-10 nm pattern into the underlying silicon substrate, to an aspect ratio of approximately 2:1. These results demonstrate the potential of exploiting SIS into carbohydrate-based polymers for nanofabrication and high pattern density applications, such as transistor devices.

## Introduction

Block copolymer (BCP) lithography has received considerable interest for the fabrication of high-resolution nanostructures due to the ability of BCP films to self-organize into regular nanometer-sized patterns over large areas.^1−6^ The BCP approach combines extremely high resolution with low-cost nanofabrication to form a variety of periodically arranged structures. Some of these structures, e.g., cylinders, lamellae, or spheres, are of great interest in device fabrication, and therefore BCPs have attracted attention for applications in nanoelectronics,^7−9^ photonics,^10^ bit-patterned media,^11^ and other technologies.^12^ In response to this interest, novel pattern transfer methods using BCP lithography in the sub-10 nm regime are needed, which is the foundation for further application development.

In BCP lithography, the size and shape of a self-assembled di-block copolymer system are mainly determined by the volume fraction of each block, the total degree of polymerization, *N*, and dissimilarity between blocks, which is described by the Flory–Huggins interaction parameter χ.^13^ Microphase separation of blocks occurs above a certain value of χ*N*, implying that higher values of χ will allow lower values of *N* to self-assemble, then resulting in smaller structures.^5,14^ In a commonly used poly(styrene)-*block*-poly(methyl methacrylate) (PS-*b*-PMMA) BCP with χ = 0.04–0.06,^5^ typical pitch of the periodic structure can be 20–50 nm, which limits the resolution to about 10 nm. Block copolymer systems with larger values of the χ parameter can, on the other hand, allow formation of tightly packed sub-10 nm structures, that are difficult to obtain by other means, e.g., photolithography or electron beam lithography (EBL). The recently developed carbohydrate-based block copolymers are examples of such high χ parameter systems, e.g., poly(styrene)-*block*-maltoheptaose (PS-*b*-MH). The high χ of this BCP is due to the hydrophilicity of the carbohydrate polymer block.^15,16^ After solvent vapor annealing (SVA), the PS-*b*-MH system is capable of forming hexagonally arranged vertical or horizontal cylinders of maltoheptaose (MH) in a poly(styrene) (PS) matrix at a pitch of 10–12 nm, achieving sub-10 nm resolution. As a consequence, this carbohydrate-based BCP is a highly promising polymer system for “single-digit” nanofabrication, provided that a suitable pattern transfer method is made available.

Directed self-assembly (DSA) of the PS-*b*-MH polymer system using a grapho-epitaxial guiding pattern made by EBL has been demonstrated by Otsuka et al.^17^ The DSA, which provides well-defined areas of highly ordered polymer domains, can be regarded as part of the patterning approach, combined with methods such as reactive ion etching (RIE) or metal deposition. Otsuka et. al also studied high-resolution pattern transfer options, based on etch selectivity of MH over PS in the CF_4_/O_2_ RIE process. Albeit, indications were that the anisotropy of the RIE process was limited and that the etch selectivity between MH and PS in vertically oriented cylindrical BCP structure was not sufficient for reliable sub-10 nm pattern transfer into the underlying Si substrate. The etch selectivity between polymer blocks can, however, be improved by a selective modification of one BCP block via diffusion of precursors that incorporate inorganic material. This type of process has been referred to in different terms, e.g., multiple pulsed vapor-phase infiltration (MPI),^18^ vapor-phase infiltration (VPI),^19^ sequential vapor infiltration (SVI),^20^ atomic layer infiltration (ALI),^21^ or sequential infiltration synthesis (SIS),^22,23^ and Leng and Losego have categorized the used nomenclature in the field.^19^ As the term SIS is widely used, we here use it to refer to the overall process. Dynamic infiltration is here referring to having a constant gas flow through the chamber and using multiple pulses of the respective precursor during each half-infiltration cycle. Another infiltration approach is to use semi-static infiltration, where the valve out of the chamber is closed before one single pulse of the precursor is introduced into the chamber. For the purging phase, the valve out of the chamber is then opened again.^24,25^

Inorganic compounds, e.g., oxides, usually have a better etch selectivity to Si than polymers do, and can therefore serve as excellent Si etch masks, facilitating high-resolution pattern transfer into underlying layers. The SIS can be implemented in a gas-phase atomic layer deposition (ALD) process, by exposing soft polymer material to vapors of precursors, e.g., trimethyl aluminum (TMA) and water for the formation of aluminum oxide, AlO*_x_*. A prolonged exposure results in diffusion of TMA molecules into the polymer and their selective reaction with available functional groups, e.g., with carbonyl moieties in PMMA for a PS-*b*-PMMA system.^23,26,27^ At the same time, the PS block will not interact with the TMA molecules, effectively resulting in selective deposition of alumina in the PMMA block only.^23^ After removal of the unaffected PS block using an oxygen plasma, the aluminum oxide mask can be used for subsequent pattern transfer. The SIS has previously been studied for infiltration into numerous polymer materials,^27−35^ but SIS into PS-*b*-MH or MH has not been covered in the literature.

Characterization of BCP structures is typically done by scanning electron microscopy (SEM), atomic force microscopy, or by X-ray scattering techniques, to determine structural data.^36^ During SIS, in situ quartz crystal microbalance gravimetry, ellipsometry, and Fourier transform infrared spectroscopy can provide information about mass uptake and reaction mechanisms.^19^ A technique complementary to the ones above is ex situ neutron reflectometry (NR), which is a nondestructive characterization technique that is perfectly suited to study properties of thin polymer films and interfaces.^37,38^ Neutrons that scatter from nuclei of the analyzed material can give information about the thickness and composition of the thin films with high spatial resolution along the depth direction.

In this paper, we report on SIS of AlO*_x_* into the PS-*b*-MH BCP system, self-assembled into either vertical or horizontal MH cylinders on a Si substrate. The thin BCP films were treated in a dedicated SIS process to selectively modify the MH block to produce an alumina mask. The process of infiltration has been characterized by SEM and NR analyses as a function of the number of SIS cycles. Here, the NR technique was for the first time applied to study the SIS process in polymers, specifically of alumina into MH and PS–OH homopolymers. This work is focused on the evaluation of dynamic infiltration of TMA and water precursors into the MH block and also includes a brief comparison of the used dynamic and semi-static SIS techniques. Finally, we demonstrate a successful dry etching process to transfer sub-10 nm features into Si, using alumina as an etch mask.

## Material and Methods

Two types of self-assembled BCP samples were prepared on top of Si substrates, with either vertical or horizontal cylinder orientation. The samples were then exposed to sequential cycles of precursors, to infiltrate one block selectively with alumina. After removing the polymer, an alumina-like pattern remained on the substrate, and the pattern was then transferred into the substrate using inductively coupled plasma reactive ion etching (ICP-RIE). Neutron reflectometry was used to analyze the effect of the infiltration process on homopolymer samples corresponding to each block in the relevant block copolymer.

### Mixture and Spin Coating

**Figure 1:**
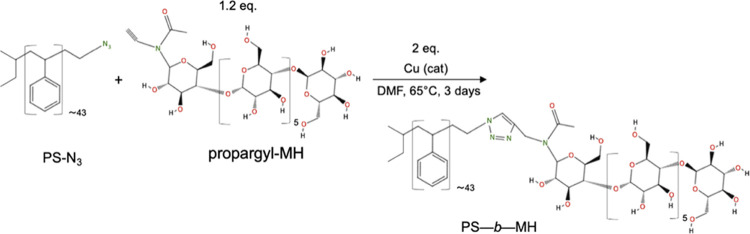
Reaction scheme for the poly(styrene)-*block*-maltoheptaose (PS-*b*-MH) block copolymer synthesis from azide-terminated polystyrene (PS–N_3_) and propargyl–maltoheptaose (propargyl–MH) using click chemistry via copper-catalyzed azide–alkyne cycloaddition.

The block copolymer was synthesized using click chemistry via copper-catalyzed azide–alkyne cycloaddition. The reaction scheme can be seen in Figure 1, and further details on polymer synthesis can be found in the Supporting Information (SI). For the BCP samples, 1.0 wt % poly(styrene)-*block*-maltoheptaose (PS-*b*-MH, 4.5 kg/mol PS, 1.2 kg/mol MH, PDI 1.06, CERMAV, two batches)^15^ was dissolved in anisole in an ultrasonic bath at approximately 40 °C for minimum 40 min. The Si substrates were pretreated on a hot plate at 200 °C for 10 min. Spin coating was made within 1 min, and samples were thereafter baked on a hot plate at 80 °C for 90 s. The polymer thickness was measured to be 12 nm for vertical cylinder samples, and 15 nm for horizontal cylinder samples. For the homopolymer preparation, 1 wt % maltoheptaose (MH, 1.2 kg/mol, Hayashibara Co., Ltd., Japan) was dissolved in deionized water and isopropanol in the volumetric ratio 3:1 and spin-coated to a layer thickness of 12 nm. The hydroxyl-terminated poly(styrene) (PS–OH, 4.5 kg/mol, PDI 1.06, anionically polymerized by CERMAV) was dissolved in anisole to 1 wt % and spin-coated to 17 nm thickness. Samples for neutron reflectivity measurements were baked on a hot plate at 80 °C for 3 min. The increased baking time was to allow removal of water from the MH films, and all samples for NR were then treated similarly. Ellipsometry was thereafter used for thickness measurements.

### Self-Assembly

The PS-*b*-MH self-assembles into hexagonally arranged MH cylinders in a PS matrix using SVA in tetrahydrofuran (THF)/H_2_O in a ratio of 4:1 (w/w).^15^ To ensure the formation of this well-defined structure, the samples were incubated in a closed container at room temperature for 60 min. The samples were then transferred to ambient conditions to kinetically entrap the self-assembled polymer.

### Sequential Infiltration Synthesis

**Figure 2:**
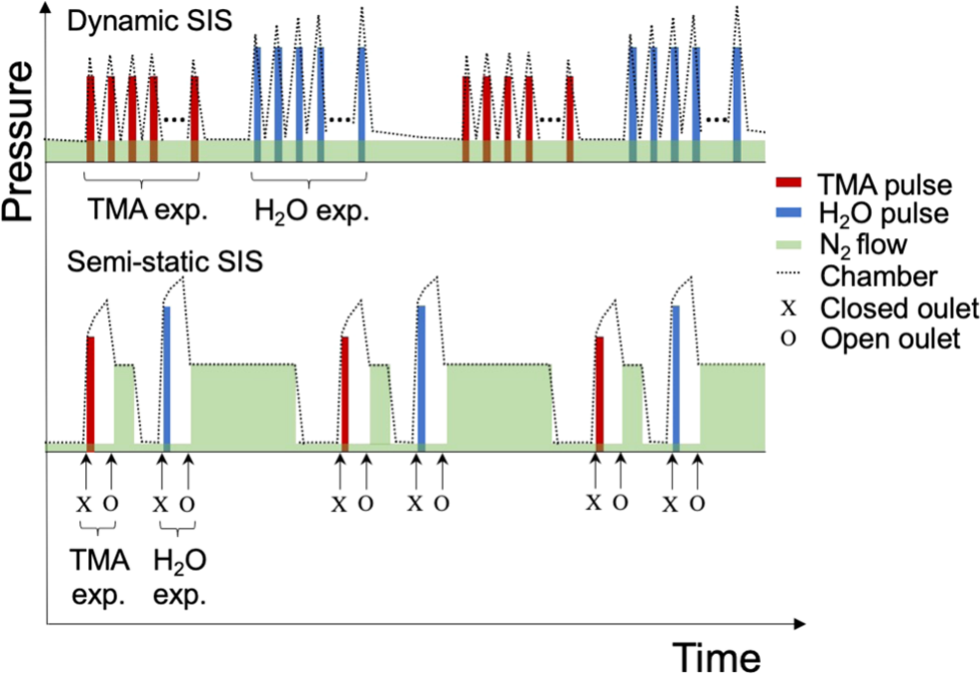
Schematic illustration of the principle of dynamic and semi-static infiltration, not to scale. Chamber pressure as a function of time.

The polymer films were infiltrated using two types of SIS processes: dynamic and semi-static. A schematic illustration of the two processes is shown in Figure 2. In the dynamic SIS process, the samples were subsequently exposed to cycles of TMA and water precursors in a Savannah S100 ALD equipment (Veeco) at 80 °C under a nitrogen (N_2_) flow of 20 standard cubic centimeters per minute (sccm).

The first part of an infiltration cycle involved a 100 s TMA precursor exposure, consisting of multiple pulses of 15 ms TMA release per second, which was followed by a 90 s N_2_ purge of the reaction chamber. The second part of the infiltration cycle involved a 50 s water precursor exposure, consisting of multiple pulses of 10 ms water release per second, and a subsequent N_2_ purge for 600 s to remove the byproducts and excess precursors.

The semi-static SIS process was performed in Savannah S100 ALD tool at the same temperature as for the dynamic process. For each precursor exposure, the nitrogen flow was set to 5 sccm, and the valve out of the chamber was closed, whereafter the precursor was introduced. For each purging step, the valve was opened to allow a flow through the chamber, and the nitrogen flow increased to 100 sccm. The first part of an infiltration cycle involved a 25 ms TMA precursor release, and a 60 s exposure time, followed by a purging step of 60 s. The second part of the infiltration cycle involved a 15 ms water release, and a 60 s exposure time, followed by a purging step of 180 s. Prior to the next cycle, the nitrogen flow was set to 5 sccm for 2 min.

### Characterization

Samples have been characterized by spectroscopic ellipsometry (M2000VI and RC2, J.A. Wollam, Co., Inc.) for layer thickness, and SEM for top-view and cross-sectional imaging. Neutron reflectometry was used for the evaluation of thickness and scattering length density of layers.

### Scanning Electron Microscopy (SEM)

SEM (SU8010, Hitachi, Ltd., Japan) was used for imaging. To enhance imaging contrast of the alumina-like pattern, the polymer matrix was typically first removed using reactive ion etching (RIE). Structures that were self-assembled into vertical cylinders, were RIE processed in Oxford Plasmalab System 100 (Oxford Instruments, United Kingdom) at 5 mTorr, in a flow of Cl_2_/Ar of 20:5 sccm, and 100 W RF for 10 s. The structures that were self-assembled into horizontal cylinders, were processed in an Apex SLR ICP-RIE (Plasma-Therm) at a pressure of 3 mTorr, using an O_2_ flow of 30 sccm, 25 W of RF power, and an ICP power of 10 W for 90 s. See the Supporting Information for further discussion regarding polymer removal.

### Neutron Reflectometry

Neutron reflectometry was performed in air using a monochromatic beam at a wavelength of λ = 5.21 Å, at the Swedish collaborative research group (CRG) instrument Super-ADAM, Institut Laue-Langevin (ILL), Grenoble, France.^39,40^ In specular reflectometry, an incident neutron beam of intensity *I*_0_ is impinging a sample surface at angle θ, and the intensity *I* of the beam reflected at the same angle is measured (see Figure S8 in the Supporting Information). The ratio *I*/*I*_0_, called reflectivity *R*, is measured as a function of *Q*_z_—a component of neutron momentum transfer perpendicular to the sample surface,^38^ where
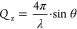
1A span of *Q*_z_, in a range from 0.005 to 0.17 Å^–1^, was achieved by scanning incidence angle θ from 0.12 to 4.0°. The raw data^40^ was first reduced by subtracting background, normalizing the reflected intensity to that of the direct beam *I*_0_, and correcting for overillumination of the sample, all using *pySAred* software.^41^ The data was thereafter analyzed using the software *GenX*,^42^ which uses Parratt recursion to simulate specular reflectivity by modeling in-depth organization of a sample. In this way, the neutron specular reflectivity profile provides information about the number and order of sublayers in the sample film, as well as parameters of each sublayer—thickness, roughness, and chemical composition. The latter can be derived from a parameter called scattering length density (SLD), which can be expressed as
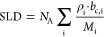
2where *N*_A_ is the Avogadro constant, ρ is the density, *b*_c_ is the neutron bound coherent scattering length, which is a unique parameter for every isotope,^43,44^ and *M* is the molar mass of the substance, summarized for all constituent elements i.^38^ The SLD can thus provide information on the included elements and on the material density. For polymers, it can also be expressed as
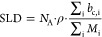
3then summarizing over the atoms included in the monomer unit.^38^ The theoretical SLDs of interest were calculated from formulas (2) and (3) and are summarized in Supporting Information Table S1. Furthermore, it follows from formula (2) that a mixture of two SLDs can be written as
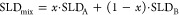
4where *x* is the volume fraction of component A, SLD_i_ is the SLD of component i, and SLD_mix_ is the SLD of the mixture. To make the assumption of a semi-infinite substrate thickness in simulations more accurate, all Si substrates for NR measurements were of 1 mm thickness. To provide information on their respective material properties, a reference measurement was made on a bare substrate, as well as on a reference sample of a 17 nm layer of atomic layer deposited AlO*_x_* on a similar substrate. An optical slab model was fitted to the data using the *GenX* software package^42^ for obtaining the thickness and SLD profile of layers.

### Pattern Transfer into Si

Samples for etching were cut into 7 × 8 mm^2^ pieces and then mounted on a sapphire carrier using a double-sided thermal tape (REVALPHA, Nitto Denko Co., Japan). The thermal tape was used to improve the thermal contact to the carrier. To remove the polymer from the AlO*_x_* infiltrated BCP pattern, oxygen plasma ashing was performed for 75 s at a pressure of 3 mTorr using an O_2_ flow of 30 sccm, 25 W of RF power, and an ICP power of 10 W in an Apex SLR ICP-RIE (Plasma-Therm). To dismount the sample after ashing, the sapphire carrier was placed on a hot plate, heated to 150 °C. The sample was then mounted on a Si carrier using the thermal tape. To etch the underlying Si, a fluorine-based recipe, a mixture of SF_6_/C_4_F_8_/Ar, was used in the same equipment as mentioned above. The Ar flow was kept constant at 20 sccm while the SF_6_/C_4_F_8_ flow ratio was changed for process optimization.

## Results and Discussion

**Figure 3:**
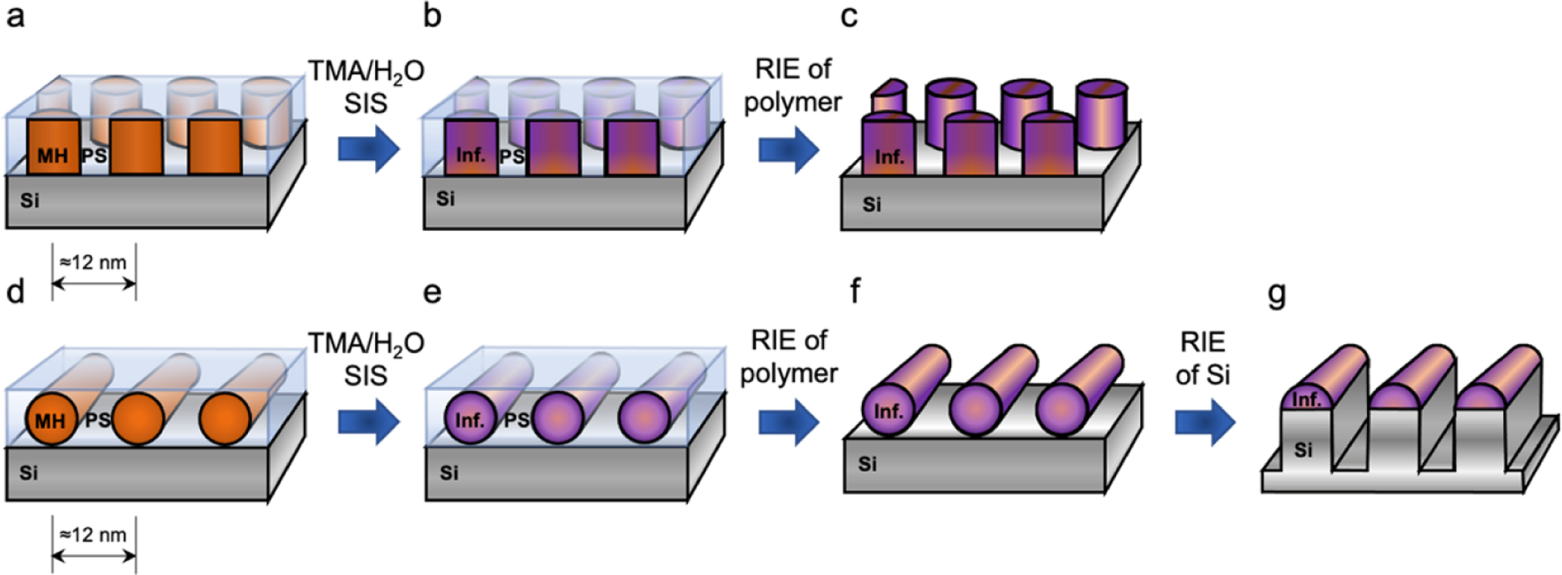
Schematic illustration of the sequential infiltration synthesis and dry etching. Top row, vertical cylinder orientation; bottom row, horizontal cylinder orientation. (a, d) Self-assembled PS-*b*-MH on a Si substrate after SVA, (b, e) after SIS of alumina into the maltoheptaose block, (c, f) the remaining alumina mask after polymer removal using reactive ion etching, and (g) the patterned silicon substrate after reactive ion etching in F-based plasma, using the alumina features as an etch mask.

In this work, we introduce SIS of alumina into PS-*b*-MH as a mean to increase dry etch selectivity between blocks. In this section, we will discuss the alumina infiltration selectivity between PS and MH and provide some insight into how the number of infiltration cycles affects dimensions and material. Finally, we demonstrate how after polymer removal, the remaining alumina-like features have been used as an etch mask for sub-10 nm pattern transfer into Si. The scheme in Figure 3 illustrates the sample preparation process, including the self-assembly, SIS, polymer removal, and pattern transfer.

### Self-Assembly

**Figure 4:**
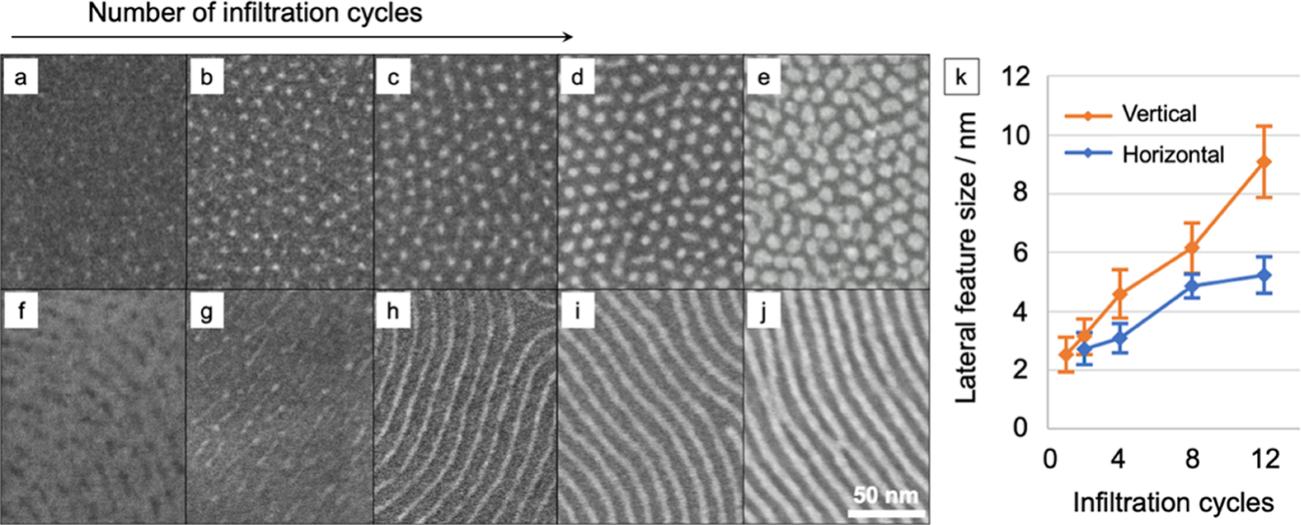
Alumina features for varying number of dynamic infiltration cycles. SEM top-view images of varying number of cycles (1–12) of sequential alumina infiltration into PS-*b*-MH, after polymer removal, showing an increase in lateral alumina feature size as a function of number of cycles. The alumina-infiltrated areas are visible as brighter features. Top row: vertical cylinders (a) after 1 cycle, (b) after 2 cycles, (c) after 4 cycles, (d) after 8 cycles, and (e) after 12 infiltration cycles; bottom row: horizontal cylinders (f) after 1 cycle, (g) after 2 cycles, (h) after 4 cycles, (i) after 8 cycles, and (j) after 12 infiltration cycles. (k) Lateral dimensions after SIS of alumina into self-assembled vertical and horizontal cylinder-oriented PS-*b*-MH, after polymer removal. Analysis was made using SEM data, and error bars represent mean value plus/minus standard deviation.

To prepare samples for SIS, PS-*b*-MH films of 12–15 nm thickness were deposited by spin coating, and then annealed to initiate self-assembly of the block copolymer. The SVA of PS-*b*-MH was carried out in a THF/H_2_O mixture at a ratio of 4:1 (w/w). For film thicknesses of 20–180 nm in this BCP system, similar SVA conditions would usually result in hexagonally arranged vertical-oriented cylindrical structures.^15^ We found that this SVA process resulted in vertical cylinders also for 15 nm film thickness from BCP batch 1. However, for 12 nm film thickness from BCP batch 2, it resulted in horizontally oriented cylinders (see Figure 4). The used annealing time is relatively short for room temperature SVA, and in general, longer annealing times improve the long-range order.^45^ On the other hand, a shorter annealing time, such as developed for PS-*b*-MH by Liao et al.,^46^ increases the throughput, which could be of importance for industrial applications.

The surface energy of the substrate is an important factor to control self-assembly. By adding a surface layer on top of the substrate the surface properties can be modified,^47^ which allows the use of different substrates. In this study, however, surface modification layers were omitted, aiming to facilitate the sequential sub-10 nm pattern transfer.

### Sequential Infiltration Synthesis

This subsection on sequential infiltration synthesis first discusses possible reaction mechanisms, then thermodynamics and kinetics. Thereafter results on the effect of number of infiltration cycles on resulting feature size are presented. Next, results from infiltration into MH, and PS respectively, are presented and discussed. This subsection also includes some results comparing the dynamic and the semi-static infiltration mode. Finally, infiltration into self-assembled BCP, and some initial results from infiltration into PS-*b*-MH are discussed.

#### Reaction Mechanisms

The reaction mechanisms in ALD and SIS are closely related. The ALD deals with deposition onto a surface, whereas the SIS deals with infiltration into a material. The reaction mechanisms for ALD using TMA and water on hydroxyl-terminated surfaces have previously been studied in detail.^48−50^ Studies of alumina infiltration into hydroxyl group-containing polymers, such as poly(vinyl alcohol),^51^ 4-aminophenol, and 4-hydroquinone,^52^ have shown that TMA reacts with the hydroxyl groups of the polymer. Yang et al. concluded in their study of phenyls, substituted with −OH, −NH_2_, or −NO_2_ groups, that whenever −OH groups were present TMA would react irreversibly with it.^52^ MH consists of three hydroxyl groups per repeating unit, or 22 per molecule (see Figure 1). It is therefore reasonable to assume that the main initial process for alumina infiltration of MH involves the reaction between TMA and these hydroxyl groups. Without experimental data on the molecular interactions during infiltration, or modeling of possible molecular energy levels, we can only speculate on the reaction mechanisms dominating for infiltration with TMA/H_2_O into PS-*b*-MH. If the interaction follows the same mechanisms as TMA/H_2_O ALD of hydroxyl-terminated surfaces, the TMA would first be adsorbed to one or two available hydroxyl groups during the TMA exposure. Unless desorbed, one aluminum atom would then covalently bond to one or to two oxygen atoms. This covalent bond would be created when the methyl (−CH_3_) groups of TMA react with the hydrogen of the hydroxyl groups, and forms CH_4_ (methane) gaseous byproduct. During the water exposure, water would then adsorb to the previously formed methyl terminated species. Unless water is desorbed, an oxygen atom of water would covalently bond to an aluminum atom. This covalent bond would be created from the reaction of one hydrogen of water and one methyl group of the previously formed methyl terminated species, and again form methane byproduct.^48−50^ The balance between the reversible complexes and direct covalent bond formation is governed by their respective energy barriers,^28,52^ and the energy gain, or loss, based on molecular interaction varies depending on participating molecules. These energy barriers are the reason that process temperature may influence the infiltration efficiency greatly. Other possible reaction mechanisms are based on Lewis adduct formation between TMA and the oxygen atom in either ether or carbonyl, followed by reactions forming covalent bonds.^26^ Each repeating unit in MH has two ether oxygen atoms, or 13 per molecule, while there is only one carbonyl group per molecule. Another possibility is the interaction between TMA and the nitrogen atoms, which occurs, e.g., for infiltration in poly(vinyl pyridine),^53^ since there are four nitrogen atoms per molecule. These functional groups are all located on the MH side of the molecule. The reaction mechanism between carbonyl and TMA has previously been studied in, e.g., PMMA,^27^ and Biswas et al. found that although the adduct formation is fast and reversible, the covalent bond formation is slow.^35^ The balance of these suggested reaction mechanisms is out of the scope of this study, but could be investigated further by, e.g., Fourier transform infrared spectroscopy or density functional theory investigations.^33^ It should be noted that apart from the functional groups available in the polymer, remaining dissolved solvents,^28^ or contaminants,^24^ may also seed the growth from their reactive functional groups.

#### Thermodynamics and Kinetics

In SIS, one should not only consider the reactivity of the functional groups, but also the dissolution and diffusion of precursors in the polymer. Waldman et al. have discussed the thermodynamics and kinetics involved in the process, and the important and controlling factors are considered to be: (1) that the precursor exposure duration and the purge time determines the distribution of the noncovalently bonded precursors, and (2) that each infiltration cycle changes diffusivity, and the precursors will likely eventually be sterically hindered by the reduction of free volume in the polymer.^28^ For an initial reaction mechanism between TMA and hydroxyl groups, the created covalent bonds are not expected to be easily broken. This is the reason that an excessive purging time is not expected to have an effect on the distribution of covalently bonded precursors. An insufficient purge time would, however, leave unreacted precursors in the material, which might lead to alumina nucleation in undesirable places.

When comparing the dynamic and semi-static SIS processes, an important difference lies in the precursor partial pressure. A schematic illustration of the principle can be seen in Figure 2. In the dynamic case, there was a constant nitrogen flow through the chamber, and each introduced TMA precursor pulse could, more or less, reset the partial pressure every second. In the semi-static case, however, the chamber outlet was closed, but with a low flow of nitrogen still entering the chamber. At the beginning of the exposure, one single pulse of precursor material was introduced. The precursor partial pressure therefore decreased with time, implying that in the case of reversible reactions, lengthy semi-static exposure times might be contra-productive.

#### Effect of Number of Cycles on Feature Size

To investigate the effect of the number of infiltration cycles on feature size, the PS-*b*-MH samples were dynamically infiltrated with different number of cycles using TMA and water. The pristine material and infiltrated samples with 1, 2, 4, 8, and 12 SIS cycles were investigated. The lateral size of the resulting alumina-like features was measured in SEM after polymer removal. A detailed discussion on polymer removal can be found in the Supporting Information. It was observed that the feature size of vertical cylinders was larger than the feature size of horizontal cylinders (see Figure 4). After four infiltration cycles, the horizontal cylinders became continuous. After 12 infiltration cycles, the lateral feature size of vertical cylinders was 9.1 nm, whereas the horizontal cylinder feature size was only 5.2 nm, and in both cases, bridging of alumina between features was occurring. After polymer removal, this bridging between features tended to bring them closer to each other, resulting in a partly disrupted structure. One possible explanation is the densification that occurs during polymer removal. An observation is that the alumina-like feature size increases with increasing number of infiltration cycles (see Figure 4). In SIS of alumina into BCP, it is expected that each infiltration cycle will increase feature size, well exceeding the thickness of one ALD cycle on hard surfaces, which is approximately 0.12 nm for AlO*_x_*.^54^ This data supports that there is infiltration into the MH block of the BCP.

#### Infiltration into Maltoheptaose (MH)

The infiltration selectivity between PS and MH is supported by the increased contrast in SEM images after polymer removal (see Figure 4). Further investigation on the infiltration selectivity, and the infiltration depth, was made by specular NR measurements of homopolymer films. We here define the infiltration depth as the total thickness of the alumina-like top layer after a certain number of infiltration cycles. The use of homopolymer films, instead of BCP films, simplified the model and the interpretation of results. The homopolymer molecular weights were the same as their respective BCP component. The investigation was performed on pristine MH, 1, 2, 4, and 8 dynamic cycles of SIS into the MH film, as well as for pristine PS–OH, 2, and 8 dynamic cycles into the PS–OH film. An NR reference measurement of a bare silicon substrate with a native oxide layer was performed using a theoretical SLD of 2.07 × 10^–6^ Å^–2^ for Si. The results showed a roughness of 2.6 Å at the Si and native oxide interface. Also, the thickness of native oxide was determined to be 5 Å with an SLD of 4.16 × 10^–6^ Å^–2^, with a roughness of 1.6 Å at the interface to air. See the Supporting Information for further details. These values were thereafter used in the following analysis. It should be noted that, in general, more than one model might be possible to fit to a given data set. For a more realistic fit, ellipsometer thickness data and SEM cross-sectional data were used to choose a consistent model.

**Figure 5:**
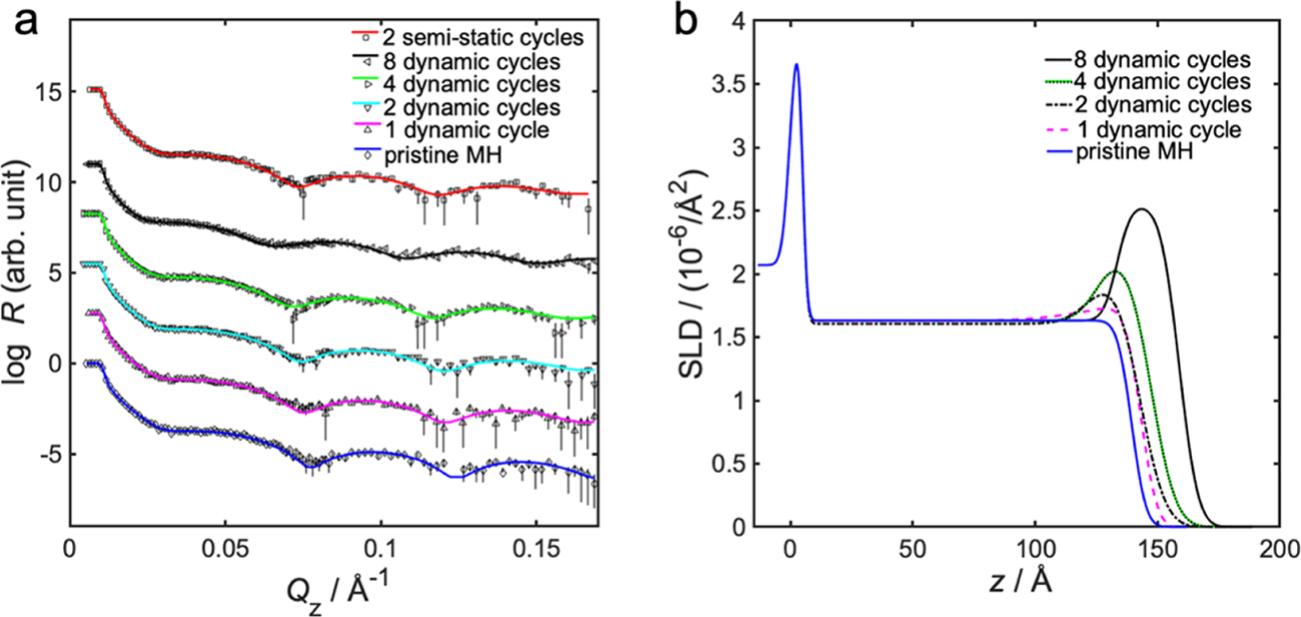
(a) Neutron specular reflectivity profiles, showing measured data points with error bars and fitted model, for infiltration into MH, and (b) fitted neutron SLD as a function of position *z* for various number of dynamic infiltration cycles into MH homopolymer. Position *z* is the distance from the supporting substrate, where *z* = 0 is the interface between the silicon substrate and the native oxide layer of 5 Å thickness. The nonmodified and modified polymer layers are positioned atop. The topmost layer is exposed to air with SLD = 0, as illustrated in the scheme given in Figure S8 in the Supporting Information.

The effects of up to eight dynamic infiltration cycles on spin-coated homopolymer MH blanket thin films were investigated by NR measurements, and the results are shown in Figure 5. The infiltration is apparent from the increase in neutron SLD for the top layer of MH (see Figure 5b), around 100–175 Å from the substrates. Moreover, NR analysis showed an infiltration depth into MH of 19–20 Å after the first two cycles, and that the total infiltration depth after eight cycles did not exceed 25 Å (see Table S5 in the Supporting Information). It should be noted that the depth error was estimated to ±3 Å. More data on the NR analysis can be found in the Supporting Information.

The neutron SLD of the dynamically infiltrated top layer of MH increased with the number of infiltration cycles, from 1.63 × 10^–6^ Å^–2^ without infiltration, to 2.54 × 10^–6^ Å^–2^ after eight cycles (see Figure 5 and Table S5 in the Supporting Information). After eight alumina infiltration cycles into MH homopolymer, the SLD difference between infiltrated top layer and noninfiltrated material was 0.91 × 10^–6^ Å^–2^. This would correspond to an inclusion of 23 vol % pure Al_2_O_3_ mixed into MH, or to 32 vol % of atomic layer deposited AlO*_x_* in MH. As a point of reference, the SLD of atomic layer deposited AlO*_x_* was measured to be 4.49 × 10^–6^ Å^–2^, which is below the theoretical value of 5.67 × 10^–6^ Å^–2^. This is reasonable, as alumina often is very porous, and the result would correspond to a 21 vol % mixture of air into pure Al_2_O_3_. The measured SLD value of 1.63 × 10^–6^ Å^–2^ was smaller than the theoretical value of 2.12 × 10^–6^ Å^–2^ for pristine MH. See the Supporting Information for further details. This difference could have more than one reason: (1) the presence of solvent in the film, which would correspond to 18 vol % water inclusion, or (2) a lower density compared to tabulated value, which would correspond to a solvent-free MH density of 1.42 g/cm^3^. Since MH is hydrophilic, one can expect some degree of water inclusion. An 18% water inclusion in carbohydrates is possible, but in the higher range for normal air humidity,^55^ which means the difference in the measured SLD could be a combination of both low density and water inclusion.

The infiltration into MH creates a dense crust of an alumina-like material close to the surface, which is assumed to sterically hinder further diffusion of precursors into the polymer. This is in line with the results from a study of TMA/H_2_O reactions within hydroxyl dense poly(vinyl alcohol) by Gong and Parsons.^26^ The precursor diffusivity therefore likely changes with the number of infiltration cycles. However, a deeper precursor diffusion is expected into polymers with fewer reactive sites,^51^ such as PS. When infiltrating the BCP PS-*b*-MH, the precursors are allowed to first diffuse through the nonreactive PS blocks, before reaching MH. For these SIS conditions, we should therefore not expect a deeper infiltration into MH than 20 Å from the PS/MH block-to-block interface. In this study, it means that MH cylinders up to 40 Å diameter inside a precursor permeable matrix should, in principle, be possible to infiltrate fully. Even if the infiltration into MH is not very deep, it is fully adequate for the formation of the small feature sizes that was the aim of this study.

Looking into the precursor solubility, using Hoy molar attraction constant contribution and tabulated values of solubility parameters (see the Supporting Information for further details), TMA appears to be more soluble in PS, whereas water is more soluble in MH. The lower solubility of TMA in MH might therefore contribute to explaining the low infiltration depth.

#### Infiltration into Polystyrene (PS)

**Figure 6:**
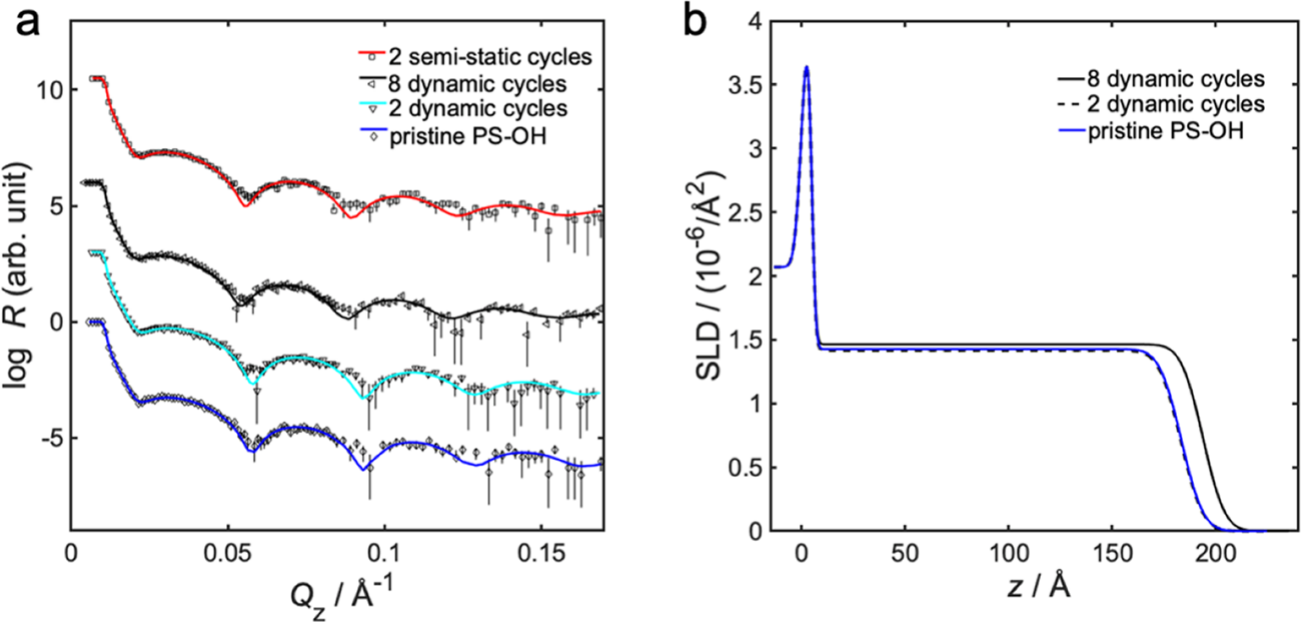
(a) Neutron specular reflectivity profiles, showing measured data points with error bars and fitted model, for infiltration into PS–OH, and (b) fitted neutron SLD as a function of position *z* for various number of dynamic infiltration cycles into PS–OH homopolymer. Position *z* is the distance from the supporting substrate, where *z* = 0 is at the interface between the silicon substrate and the native oxide layer of 5 Å thickness. The polymer layer is positioned atop and is exposed to air with SLD = 0, as illustrated in the scheme given in Figure S8 in the Supporting Information.

Cianci et al. have previously found that TMA and water can diffuse through pure PS, and that there can be a high infiltration selectivity between PMMA and PS for a low number of infiltration cycles, unless the PS includes defects.^24^ To further investigate the dynamic infiltration selectivity between MH and PS, but at the same time investigate the diffusion through PS, a hydroxyl-terminated PS (PS–OH) was examined, meaning that each PS molecule now included one reactive site for the TMA precursor. Neutron reflectivity data showed that the SLD of PS–OH was 1.43 × 10^–6^ Å^–2^ without infiltration, and increased by 0.03 × 10^–6^ Å^–2^ after eight cycles (see Figure 6 and Table S4 in the Supporting Information). The difference between the measured and the theoretical SLD value for pristine PS–OH would correspond to a somewhat higher density of 1.06 g/cm^3^, which lies within the expected span. The slightly higher SLD after infiltration could be a result of a reaction between the hydroxyl groups and TMA, initiating alumina infiltration. This result would correspond to an evenly distributed inclusion of 0.8 vol % pure Al_2_O_3_ in the PS–OH after eight infiltration cycles. However, it should be noted that this small increase is in the same order as the measurement error. If there is an actual increase, this is an indication that in the used SIS process, the TMA and water precursors do diffuse through the entire PS–OH film of 17 nm thickness, and that the infiltration selectivity between MH and PS–OH is high. It should also be noted that the infiltration selectivity between MH and PS without hydroxyl termination should be even higher, as it lacks reactive sites for TMA. Additional information on the NR analysis can be found in the Supporting Information.

#### Dynamic versus Semistatic Infiltration

A brief comparison between the dynamic and the semi-static infiltration techniques has been performed using two semi-static infiltration cycles into MH and PS–OH homopolymer film, respectively. The NR measurement data confirmed that the infiltration depth into MH after two semi-static cycles was 3 Å deeper than the depth after two dynamic cycles, and equal to the depth after four dynamic cycles (see Table S5 in the Supporting Information, Figures 5a and7a). However, the SLD after two semi-static cycles was 0.13 × 10^–6^ Å^–2^ lower than the SLD after two dynamic cycles. It should be noted that these differences are in the same order as the measurement error. For 2-cycle infiltration into PS–OH, the SLD was essentially the same for both dynamic and semi-static infiltration (see Table S4 in the Supporting Information, Figures 6a and 7b). The Supporting Information provides further details. Thus, there were indications of differences in performance between the two techniques. The semi-static infiltration method used about 60 times less TMA precursor material than the dynamic, but was still performing in a comparable manner. Based on the indication of a deeper infiltration, incorporating less AlO*_x_* material, in the semi-static SIS process, compared to the dynamic, one interpretation could be that a lower precursor partial pressure could be promoting deeper infiltration, in the case where the reactive functional group density within the material is very high. Steric hindrance would then likely occur after a larger number of infiltration cycles. It is possible that further investigation of the influence of process temperature, precursor partial pressure, precursor exposure times, and purging times might further increase the possible infiltration depth and/or alumina content of the infiltrated material. This is, however, out of the scope of this study.

**Figure 7:**
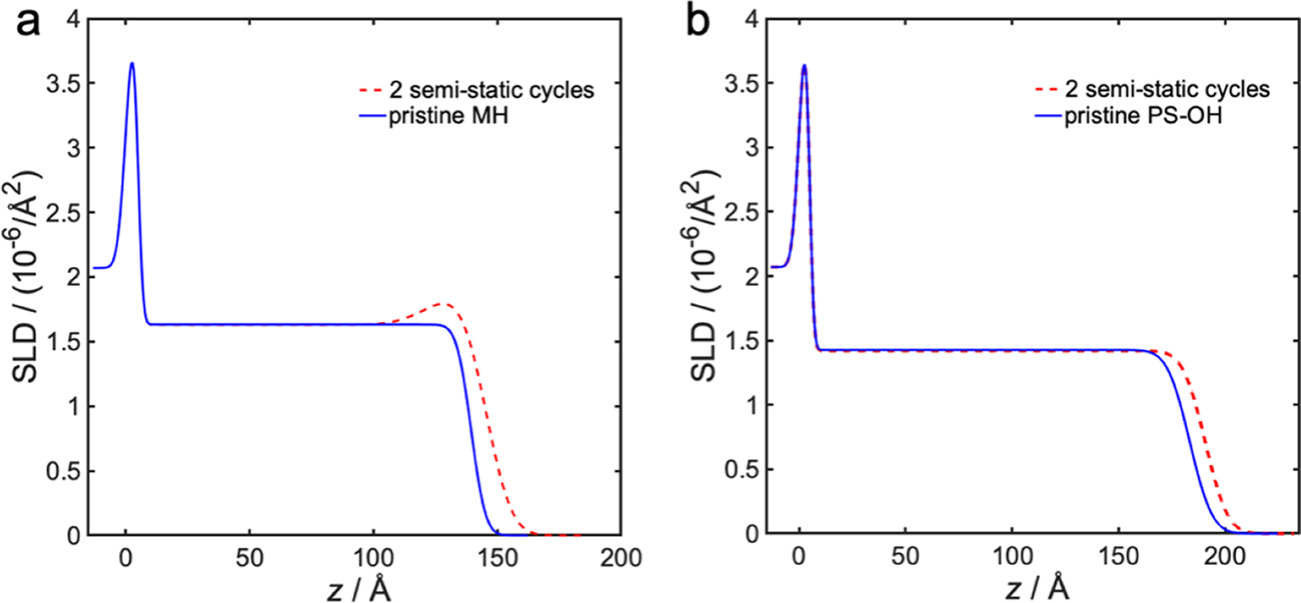
Fitted neutron SLD profile as a function of distance, *z*, from the substrate surface for semi-static infiltration cycles into (a) MH homopolymer, and (b) PS–OH homopolymer. Position *z* is the distance from the supporting substrate, where *z* = 0 is at the interface between the silicon substrate and the native oxide layer of 5 Å thickness. The polymer layer is positioned atop and is exposed to air with SLD = 0 as illustrated in the scheme in Figure S8 in the Supporting Information.

#### Infiltration into PS-*b*-MH

In this study, SIS of TMA and water into PS-*b*-MH with molecular weight 4.5k-*b*-1.2k was investigated. If pattern density is to be increased, the molecular weight of the BCP should be lowered. However, the BCP molecular weight needs to be high enough to obtain sufficiently large regions of ordered self-assembly, and the precursor molecular weight low enough not to hamper diffusive transport. The diffusion in nanoscale BCP morphology differs from that in a homopolymer. The diffusivity in both homopolymer and BCP, however, decreases with increasing degree of polymerization *N*, especially for low *N*.^56^ In our experiment, *N* for MH was 7, and for PS–OH, it was approximately 43, and therefore it can be assumed that the diffusivity in this regime is very sensitive to molecular weight. The homopolymer molecular weights were therefore chosen to be equal to their respective blocks in the BCP.

Models of small-molecule diffusion in BCP, having very different homopolymer diffusivity for respective block, show that the constraints imposed by the morphology lower the diffusivity, compared to the diffusivity without constraints.^56,57^ Especially, the diffusivity in the direction parallel to the cylinders is higher than the diffusivity in the direction perpendicular to the cylinders.^56^ Although the situation in our study might not be accurately modeled by a BCP bulk situation, these models could contribute to explaining any differences in diffusion in BCP when cylinders are oriented horizontally, and vertically, respectively.

Initial results from a study reported elsewhere,^58^ including NR analysis of TMA/H_2_O infiltration into the BCP system PS-*b*-MH with molecular weight 4.5k-*b*-1.2k indicate that the Al_2_O_3_ content of the BCP is 2.4 vol % after four dynamic infiltration cycles, which is 18% of the Al_2_O_3_ content in MH homopolymer after the same treatment. As the volumetric fraction of MH in the BCP is 19%, using the experimental densities found in this study (1.42 g/cm^3^ for MH, and 1.06 g/cm^3^ for PS), we conclude that the initial results from NR analysis of TMA/H_2_O infiltration into the BCP system PS-*b*-MH are in line with the homopolymer infiltration results presented here.

This infiltration study shows that eight dynamic infiltration cycles can create well-defined, alumina-like features of 4.9 nm diameter at the positions of horizontal maltoheptaose cylinders in the self-assembled 12 nm pitch block copolymer PS-*b*-MH. These samples were used for further investigations of the pattern transfer.

### Pattern Transfer into Si

This SIS-prepared alumina-like pattern was used as an etch mask for pattern transfer. In this experiment, the horizontal cylinder pattern was used to transfer the pattern into the underlying Si substrate using ICP-RIE. As the first etch step, polymer was removed in an oxygen plasma. The O_2_ plasma process time was varied between 1 and 4 min. It was observed that 1 min was at the border of what was required to remove the entire PS block, but 75 s was enough to clear the features and reveal the Si surface. To conduct Si etching, a pseudo-Bosch process was used.^59^ In this approach, an etchant (SF_6_) and a passivation gas (C_4_F_8_) were simultaneously injected into the chamber, in combination with an inert gas (Ar). This normally ensures an anisotropic etching with smooth sidewalls. However, the etch rate was found to be lower than other approaches like Bosch or cryogenic etching.^60,61^ As expected, the etching of such small features was different than of large features. The etch rate was low (around 10 nm/min), which in turn limits the possible aspect ratio and etch selectivity to lower numbers, compared to what has previously been reported for Si etching of larger features using alumina mask.^59^ Improvement of etch selectivity and etch rate might be possible by replacing the used pseudo-Bosch process with cryogenic etching or a hydrogen bromide-based etch recipe.^62,63^ The RF and ICP powers were varied, and results proved that etching of the SIS prepared BCP samples was very sensitive to these parameters. A slight increase in the used values of these parameters degraded the etch selectivity significantly. The estimated value for the etch selectivity of Si over alumina-like infiltrated PS-*b*-MH mask was approximately 2:1, which is lower than previously indicated values for SIS masks fabricated by different types of BCPs.^62^ However, it must be noted that the pattern size, as well as the etch recipes were different than in this study and a direct comparison is therefore not straightforward. It was also observed that by increasing the etch time, the mask erosion speeded up nonlinearly. It could imply that the mask had a density gradient from top to bottom. To increase etch selectivity, the etch rate of the semiconductor has to improve, while the mask consumption should stay the same or decrease. As the process was very sensitive to RF power, the etch rate was controlled by changing the gas ratio. By increasing the SF_6_ flow from 33 to 44% (out of total flow of SF_6_ + C_4_F_8_) the etch rate was increased without significant change in mask etching or degrading the profile anisotropy.

**Figure 8:**
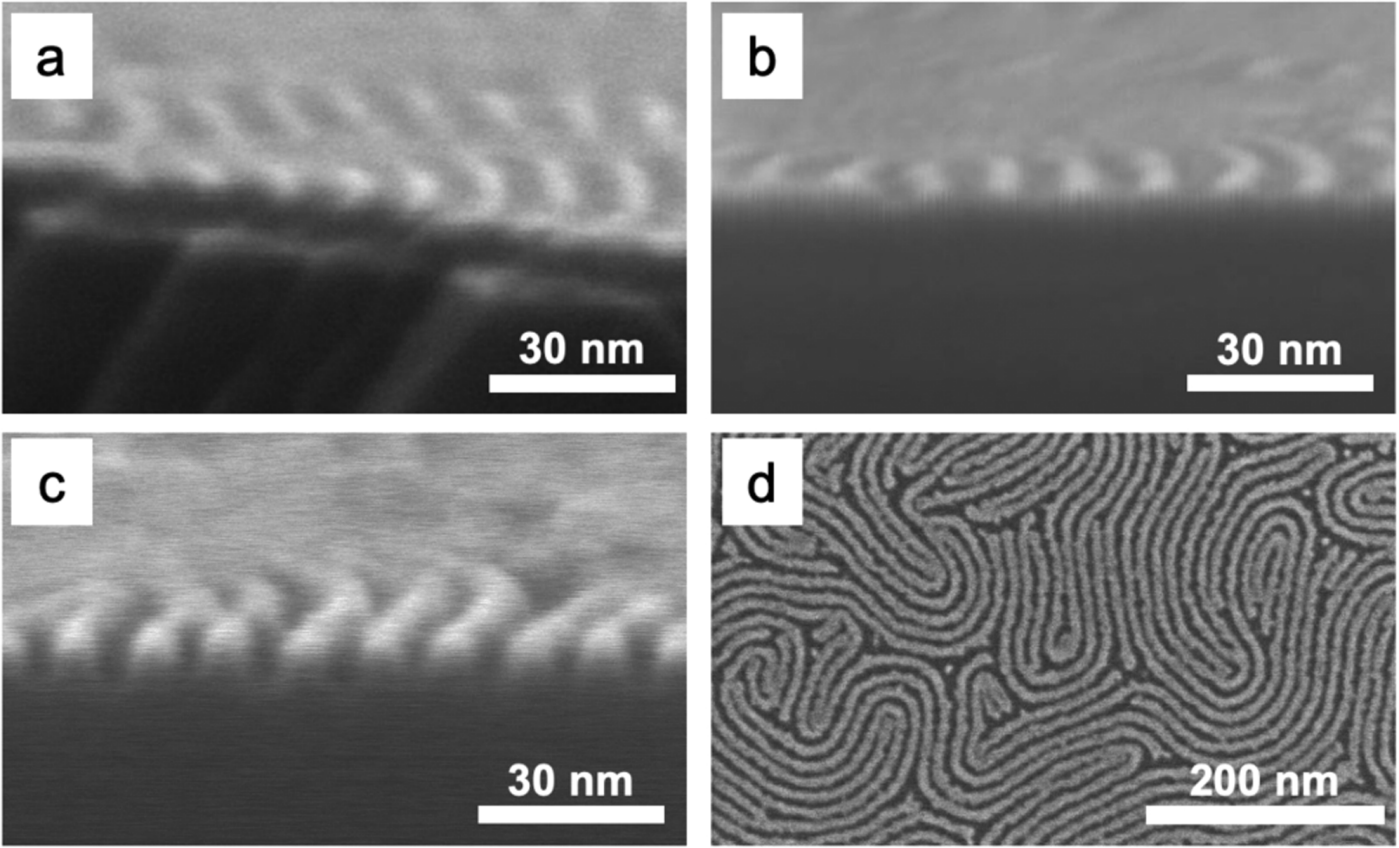
Tilted cross-sectional SEM images of eight-cycle dynamically infiltrated horizontal cylinder PS-*b*-MH, (a) before polymer removal, (b) after polymer removal, and (c) after pattern transfer into Si. (d) Top-view SEM image after pattern transfer into Si.

Figure 8 shows SEM images of infiltrated BCP samples before and after 1 min polymer removal, as well as after pattern transfer into Si using 5 mTorr pressure, 25 W RF power, 300 W ICP power, and a gas flow of SF_6_/C_4_F_8_/Ar of 26/54/20 sccm for 45 s, measuring resulting trench dimensions to approximately 5 nm in width and 10 nm in depth. The aspect ratio of critical dimension is, thus, approximately 2:1.

## Conclusions

In this study, both dynamic and semi-static SIS into MH and PS–OH polymer were investigated by ex situ NR. This characterization method provided a quantitative measure of the infiltration depth of TMA and water into MH homopolymer. The results showed an increase in neutron SLD with the number of infiltration cycles. This increase was then correlated to a percentage of Al_2_O_3_ included in the alumina-like infiltrated layer. Neutron reflectometry also showed selective infiltration into MH over PS–OH, as well as indicated infiltration to the full depth of the PS–OH film. When comparing the two SIS methods, they were found to perform in a comparable manner although the semi-static infiltration method used about 60 times less TMA precursor material. Furthermore, ex situ SEM investigation of dynamic SIS of TMA and water into 12 nm pitch PS-*b*-MH showed selective infiltration into the MH cylinders. The feature size of the alumina-like horizontal cylinders increased from 3.1 to 5.2 nm when the number of dynamic cycles changed from 4 to 12. Alumina-like horizontal cylinders were then used as a dry etch mask, and after polymer removal, the sub-10 nm pattern was successfully transferred into the underlying Si substrate using ICP-RIE. These results show that carbohydrate-based BCP lithography, in combination with SIS, is a viable route for pattern transfer in single-digit semiconductor nanofabrication. One suitable application could be DSA to improve line and space resolution in logic devices, e.g., in fin field-effect transistors (FinFETs). Utilizing the method presented here, the fin pitch could potentially be decreased to 12 nm, which is far denser than the production prediction of 24 nm fin pitch for the so-called 3 nm node technology.^8^

## Supporting Information Available

Polymer synthesis; self-assembly; solubility; polymer dry etching; and neutron reflectometry (PDF)
