# Supplementary material for: Poly(styrene)-block-Maltoheptaose Films for Sub-10 nm Pattern Transfer: Implications for Transistor Fabrication
Source: ACS Appl Nano Mater. 2021 May 13;4(5):5141–51. doi: 10.1021/acsanm.1c00582 (PMC8290925; doi:10.1021/acsanm.1c00582)
Supplement: Supplementary file 1 — an1c00582_si_001.pdf [file an1c00582_si_001.pdf]

Supporting Information

Poly(styrene)-*block*-Maltoheptaose Films for Sub-10 nm Pattern Transfer: Implications for Transistor Fabrication

Anette Löfstrand,*[a] Reza Jafari Jam,[a] Karolina Mothander,[b] Tommy Nylander,[b] Muhammad Mumtaz,[c] Alexei Vorobiev,[d] Wen-Chang Chen,[e] Redouane Borsali [c] and Ivan Maximov*[a]

[a] NanoLund and Solid State Physics, Lund University, SE-221 00 Lund, Sweden
[b] NanoLund and Physical Chemistry, Lund University, P.O. Box 124, SE-221 00 Lund, Sweden
[c] Univ. Grenoble Alpes, CNRS, CERMAV, 38000 Grenoble, France
[d] Division for Materials Physics, Department of Physics and Astronomy, Uppsala University, P.O. Box 516, SE-751 20 Uppsala, Sweden
[e] Advanced Research Center for Green Materials Science and Technology, National Taiwan University, Taipei 10617, Taiwan

* e-mail: anette.lofstrand@ftf.lth.se, ivan.maximov@ftf.lth.se

The supporting information includes experimental details on polymer synthesis, polymer characterization using proton nuclear magnetic resonance (^{1}H NMR) spectroscopy and size exclusion chromatography (SEC), characterization of self-assembled PS-*b*-MH layers using atomic force microscopy (AFM) and scanning electron microscopy (SEM), and a discussion regarding reactive ion etching (RIE) processes used for polymer removal after sequential infiltration synthesis (SIS). It also contains further detail on the neutron reflectometry (NR) analysis included in the paper, as well as solubility estimations of precursors in polymers.

Polymer synthesis

Material

p-toluenesulfonyl chloride (*regentPlus*, ≥99%), NaN_3 (*regentPlus*, ≥99.5%), trimethylamine (TEA, 99%), 1,4-dioxane (anhydrous, 99.8%), and calcium hydride (CaH_2, 95%), magnesium sulfate (anhydrous, *regentPlus*, ≥99.5%), ethylene oxide solution (2.5-3.3M in THF), di-*n*-butylmagnesium solution (1.0 M in heptane) and *sec*-butyllithium solution (1.4 M in cylcohexane) were purchased from Sigma Aldrich and used as received. Toluene (Biosolve) was first distilled over CaH_2 and then over polystyryllithium. Styrene (Sigma Aldrich, *ReagentPlus,* ≥99%) was first distilled over CaH_2 and then over di-n-dibutylmagnesium. CH_2Cl_2 (CP) stabilized by amylene and pyridine (extra dry) was obtained from Biosolve and distilled over CaH_2 at 35°C prior to use. Maltoheptaose (MH) (Hayashibara Company) alkynyl group according to literature method.[1] The Cu/CuO nanopowder (CuNP, 20-50 nm, 99.9% metal basis) was purchased from Alfa Aesar. Tetrahydrofuran (THF), absolute methanol and absolute ethanol were bought from Biosolve. And *N*,*N*-dimethylformamide (DMF, Fisher scientific) were used as received. Milli-Q water was obtained by water purification to a resistivity of 18.2 MΩ cm using a Millipore Ultrapure system. The deuterated solvents were purchased from Eurisotop. The cuprisorb resin was bought from Seachem.

Synthesis of propargyl-maltoheptaose (propargyl-MH)

N-maltoheptaosyl-3-acetamido-1-propyne (propargyl-MH) was synthesized according to previous report.[1]

Synthesis of hydroxyl-terminated poly(styrene) (PS-OH)

Hydroxyl-terminated poly(styrene) was prepared by anionic polymerization of styrene accompanied by the termination with ethylene oxide. Toluene (300 mL) was introduced in a 1 L flamed dried round bottom two-necked flask equipped with magnetic stirrer and specially designed joint with roto-flow, under vacuum. Styrene (40 g, 44.15 mL) was then added and the flask filled with argon. Sec-butyllithium (~1.4 M in cyclohexane, 10 mM, 7.14 mL) was then introduced in order to initiate the polymerization. The color of the reaction mixture turned red. The reaction flask was placed in an oil bath at 35°C for 3h. The polymerization reaction was finally terminated by the addition of ethylene oxide (5.0 mL, ~3M solution in THF) in the reaction mixture, accompanied by the addition of excess of degassed methanol. The solvent was removed under vacuum using rotary evaporator at 40°C. The polymer was redissolved in appropriate amount of THF and precipitated twice in methanol (1L). The white precipitate of hydroxyl terminated poly(styrene) was filtered using a sintered glass funnel under vacuum and then dried in a vacuum oven at 40°C overnight. The outcome was 39 g of solid product, 95% yield. The sample was characterized using ^{1}H NMR and SEC, which resulted in M_n (^{1}H NMR) ~ 4500 g/mol, and M_n (SEC, DMF) = 3800 g/mol (see **Figure S1** and **Figure S3**).

Synthesis of azido-functionalized poly(styrene) (PS-N_3)

The azido-functionalized poly(styrene) was prepared in following two steps: In the first step, poly(styrene) (10.00 g, 2.22 mM, M_n = 4500 g mol^{-1}) was dissolved in dried dichloromethane (100 mL) in a two-necked round bottom flame-dried flask equipped with magnetic stirrer, followed by addition of trimethylamine (9.3 mL, 66.7 mM). The temperature of the reaction was reduced to 0 °C by putting the flask in an ice bath. Finally, *p*-toluenesulfonyl chloride (4.24 g, 22.2 mM) was added in small portions under argon flow. The temperature of the system was allowed to raise slowly to room temperature and the reaction mixture was allowed to react for overnight under stirring. The reaction mixture was diluted by the addition of CH_2Cl_2 (100 mL) and transferred into separating funnel (500 mL) where residual salts were removed by extraction with water (3×100 mL). The organic layer was then dried using $MgSO_4$ and the solvent was removed by rotary evaporator. The polymer was redissolved in appropriate amount of THF and precipitated twice in methanol (500 mL). The white precipitate of tosyl terminated poly(styrene) (PS-OTs) was filtered using a sintered glass funnel under vacuum and dried in a vacuum oven at 40°C overnight. The outcome was 9.3 g of solid product, 90% yield. The sample was analysed by ^{1}H NMR (see **Figure S1**).

In the second step, ω-tosyl polystyrene (9 g, 1.94 mM) prepared in above step was charged in a two-necked round bottom flask containing DMF (60 mL) and equipped with magnetic stirrer. NaN_3 (2.50 g, 38.7 mM) was then added under stirring and the reaction mixture was placed in an oil bath at 60°C overnight. The system was then let to coole down to room temperature, diluted with CH_2Cl_2 (200 mL) and transferred into a separating funnel where it was repeatedly washed with water to remove the residual tosylate salt, excess of NaN_3 and DMF. The organic

layer was then dried by adding anhydrous $MgSO_4$. CH_2Cl_2 was removed by evaporation using rotary evaporator. The polymer was redissolved in an appropriate amount of THF and precipitated twice in methanol (500 mL). The white precipitate of azido-terminated poly(styrene) was filtered using a sintered glass funnel under vacuum and dried in a vacuum oven at 40°C overnight. The outcome was 8.0 g of solid product, ~89% yield. The sample was characterized by ^{1}H NMR and SEC (see **Figure S1** and **Figure S3**).

Synthesis of poly(styrene)-*block*-maltoheptaose (PS-*b*-MH) block copolymer

Poly(styrene)-*b*-maltoheptaose was prepared by click chemistry of azido-functionalized PS and alkynyl-functionalized maltoheptaose. In a round bottom, one-necked flask equipped with roto-flow and magnetic stirrer was charged with ω-azido poly(styrene) (1 eq, 6.0 g, ~1.33 mM), propargyl-maltoheptaose (1.2 eq, 2.0 g, 1.6 mM), and DMF (40 mL) and degassed by three freeze-pump-thaw cycles. Thereafter, copper nanopowder (2 eq vs acetylene group, 205 mg, 3.20 mM) was added to the solution under argon flow and subjected to another freeze-pump-thaw cycle. The solution was stirred under argon atmosphere at 65°C for 3 days. At the end of the reaction, the crude heterogeneous solution was diluted with THF and filtered through diatomaceous earth. The obtained filtrate was stirred with 5.0 g cuprisorb resin at 40°C overnight. The solution was filtered to remove the cuprisorb resin and the solvent was removed by distillation using roto-evaporator. The crude product was redissolved in appropriate amount of THF and precipitated in methanol to remove excess of maltoheptaose. The unreacted poly(styrene) was removed by re-precipitation of block copolymer in cyclohexane/heptane (60/40, v/v) mixture. The resulting white solid was dried in vacuum at 40°C overnight and characterized by ^{1}H NMR and SEC (see **Figure S2** and **Figure S3**). The outcome was 6.5g solid product, ~85% yield.

Polymer characterization

^{1}H NMR spectra of polymer samples were recorded on a Bruker Avance 400 MHz spectrometer with a frequency of 400.13 MHz and calibrated with the signal of deuterated solvent (**see Figure S1** and **Figure S2**). The size exclusion chromatography (SEC) was performed at 40°C using an Agilent 390 MDS system (290 LC pump injector, ProStar 510 column oven, 390 MDS refractive index detector) equipped with Knauer Smartline UV detector 2500 and two Agilent Poly Pore PL1113−6500 columns (linear, 7.5 × 300 mm; particle size, 5 µm; exclusion limit, 200−2,000,000) in DMF containing lithium chloride (0.01 M) at the flow rate of 1.0 mL min^{-1} (see **Figure S3**).

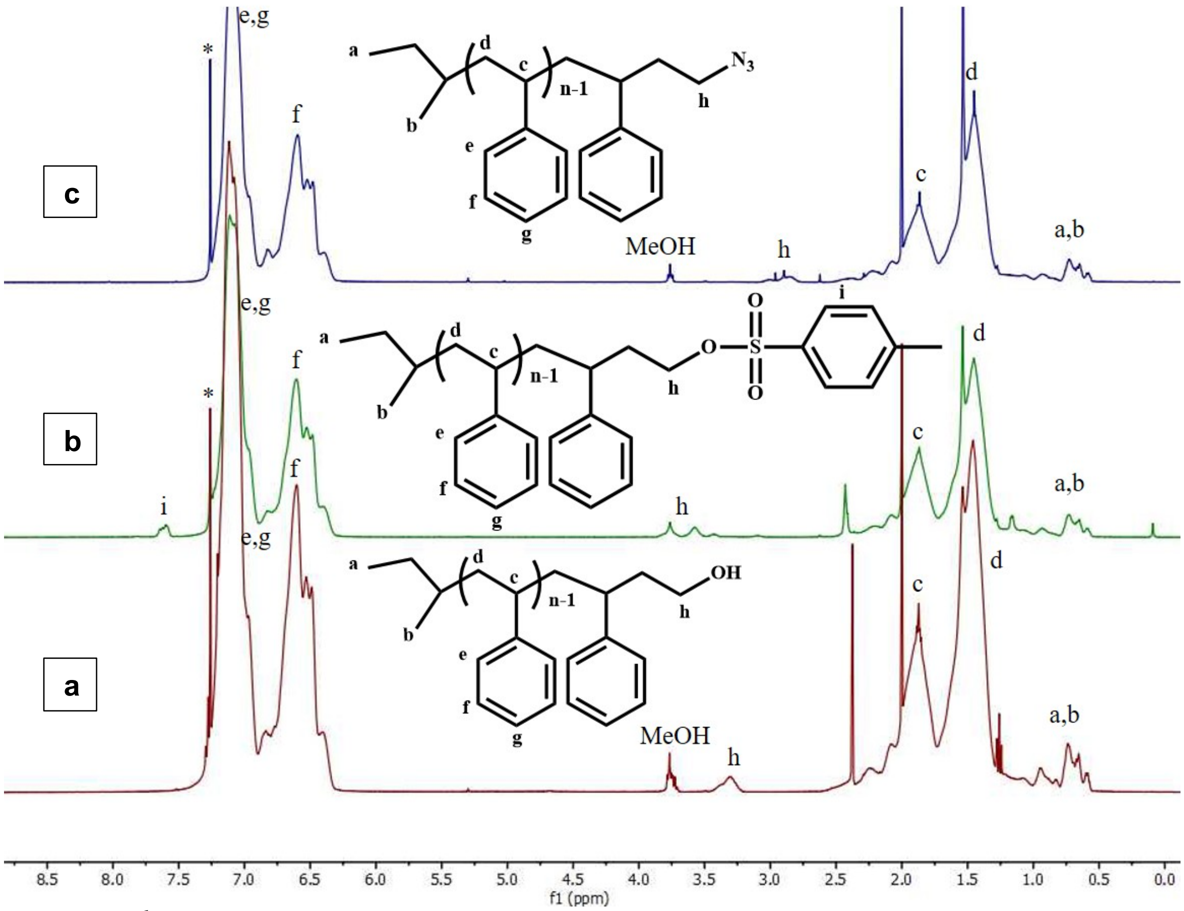

Figure S1. *^{1}H NMR of a) PS-OH, b) PS-OTs, and c) PS-N_3 in $CDCl_3$ at 25°C (400 MHz).*

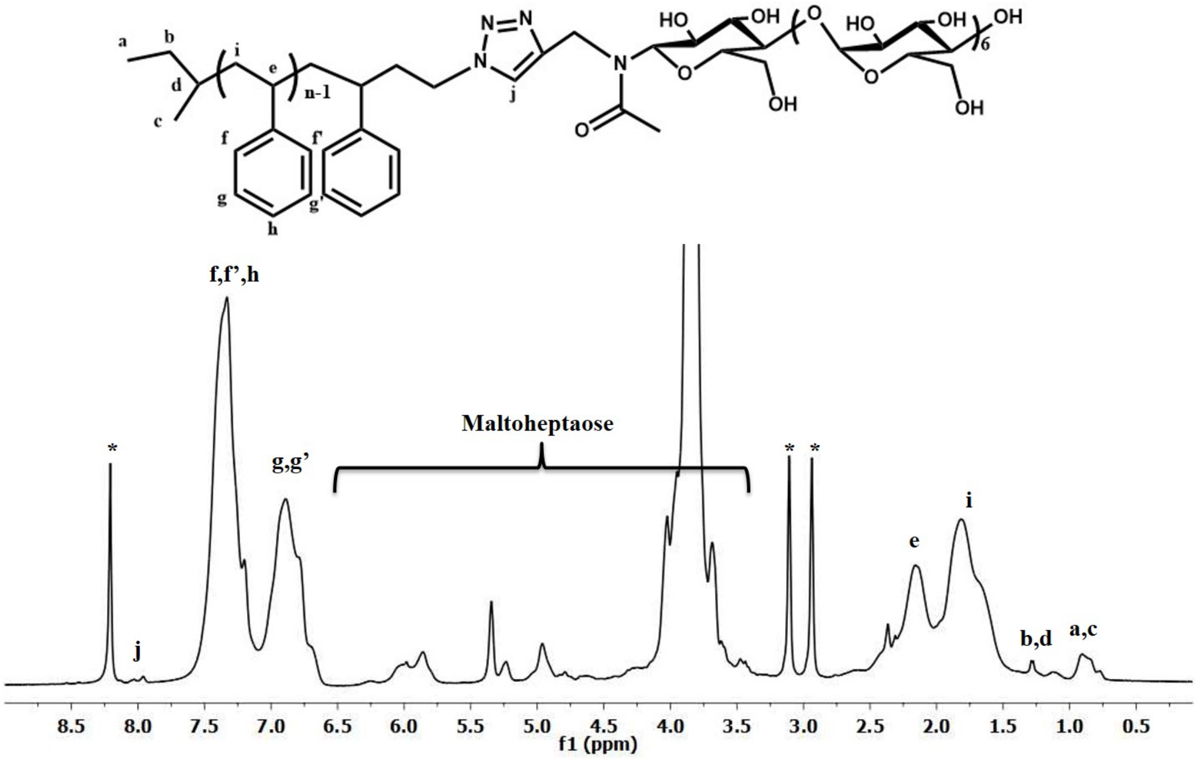

Figure S2. *^{1}H NMR of PS-b-MH in DMF-d7 at 25°C (400 MHz).*

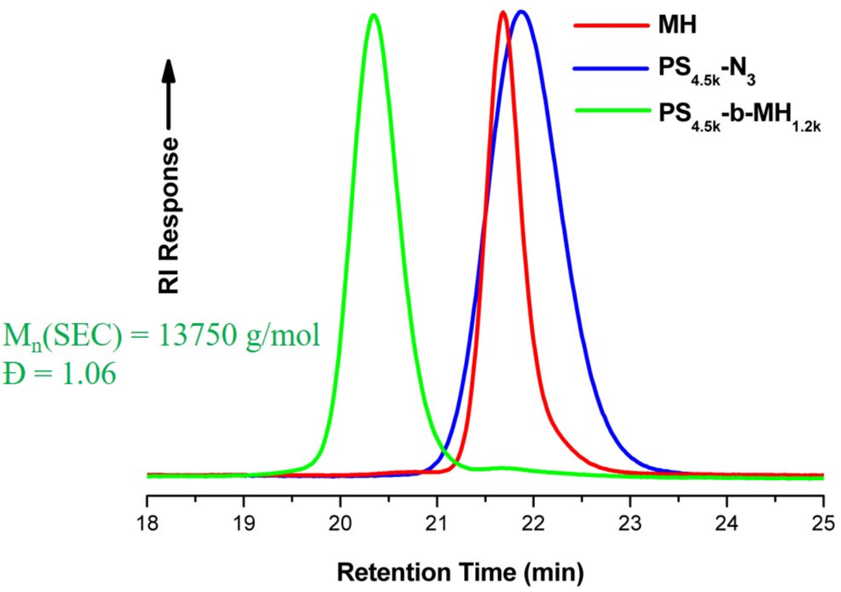

Figure S3. *SEC traces of $PS_{4.5k}$-N_3 (Blue), $MH_{1.2k}$ (Red) and $PS_{4.5k}$ -b-$MH_{1.2k}$ (green) using DMF as an eluent and PS calibration at 40°C.*

Self-assembly

Characterization of self-assembled, horizontal cylinder oriented, PS-*b*-MH using AFM in tapping mode in an Icon (Bruker, US), can be seen in **Figure S1**. The height difference can be interpreted as an existence of two self-assembled layers, in some areas. The height difference between the two self-assembled layers of vertical cylinders was AFM characterized in a Dimension 3100 (Bruker, US), and measured to be 7.7 nm (see **Figure S5**).

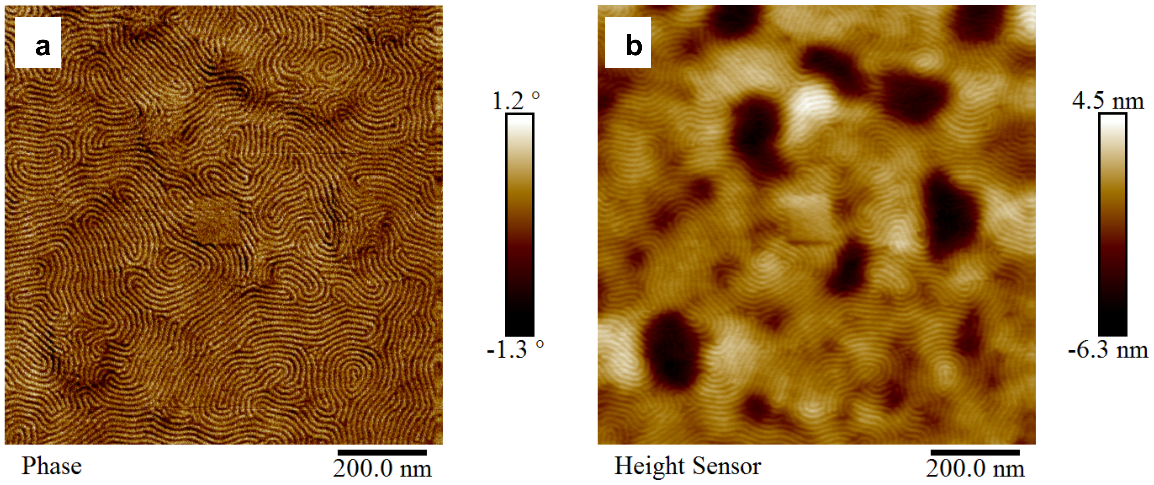

Figure S4. *AFM images of PS-b-MH. a) phase image, and b) height sensor image, showing horizontal cylinder arrangement. Brighter lines represent the MH block.*

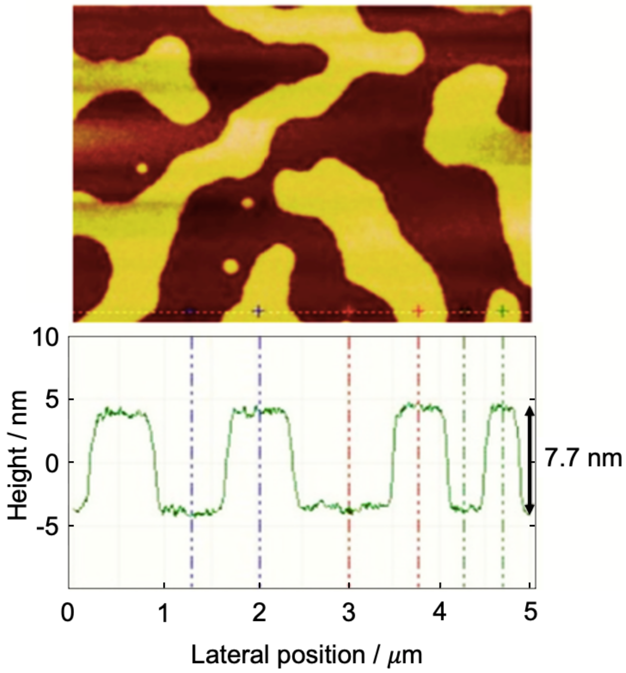

Figure S5. *Vertically self-assembled $PS_{4.5k}$-b-$MH_{1.2k}$. AFM section analysis height data of a 5x5 µm^2 scan with 15 nm initial thickness, showing a height difference of 7.7 nm from the lower plateau (showed in brown) to the upper plateau (the islands, showed in yellow).*

In SEM, the secondary electron contrast between the pristine blocks, PS and MH, was insufficient for a reliable detection of self-assembly. Therefore, the BCP self-assembly structures were imaged by SEM after an 8-cycle dynamic SIS process and polymer removal. Characterization of self-assembled, horizontal and vertical cylinder oriented, PS-*b*-MH using SEM (SU8010, Hitachi, Ltd., Japan), can be seen in **Figure S6**.

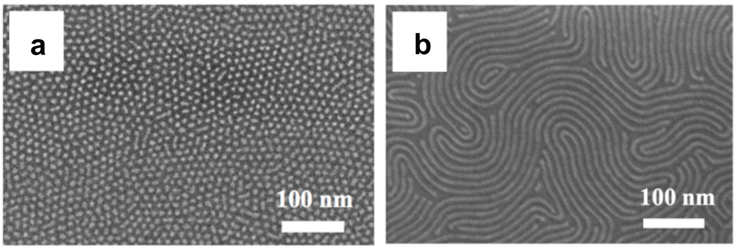

Figure S6. *Top view SEM images of a) vertically oriented cylinders, and b) horizontally oriented cylinders of self-assembled, sequentially infiltrated PS-b-MH after polymer removal.*

Polymer removal

Optimizing the reactive ion etching (RIE) process to etch the polymer from the infiltrated BCP samples was not straight forward. Polymer removal was first explored on 8-cycle infiltrated PS-*b*-MH with vertically oriented cylinders, using RIE in an Oxford Instruments Plasmalab System 100 in 50 sccm O_2 at 10 mTorr, 10 W RF and 600 W ICP for 15 s. SEM inspection showed that the alumina-like features were disrupted from their positions (see **Figure S7**). When instead a low-pressure polymer removal was performed using reactive ion etching in the same system in Cl_2/Ar (20:5) sccm at 5 mTorr and 100 W RF for 10 s, the features remained in

their positions. The latter process was therefore used for evaluation of lateral size of the alumina-like features of the vertical cylinders. The etch rate of alumina should be slower in an oxygen plasma than in a chorine-based plasma. As there was a concern that the chlorine would also etch the formed alumina-like mask, the process was further optimized, for the horizontal cylinder samples, in an Apex SLR ICP-RIE (Plasma-Therm, US), allowing lower pressure oxygen plasma processes. Since the etch time in chlorine-based plasma is short, and the measured lateral sizes of vertical cylinders are actually larger than for horizontal cylinders, it is here considered valid to compare the measured values after polymer removal, using the two methods. However, indications are that the lateral feature size is approximately 0.9 nm, or 15%, smaller after the chlorine-based plasma process, than after the initial oxygen-based plasma process (see **Figure S7**).

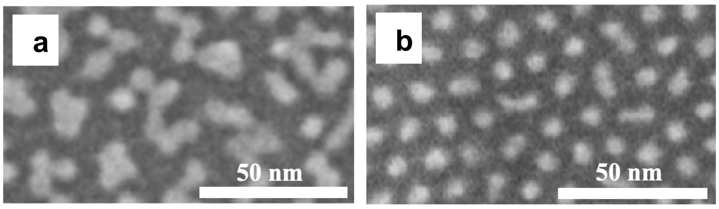

Figure S7. *SEM top view images of 8-cycle dynamically infiltrated PS-b-MH. a) after higher pressure oxygen plasma polymer removal, showing the displacement of the alumina-like features, and b) after using low pressure Cl_2/Ar polymer removal.*

Neutron reflectometry (NR)

Above the critical Q_z for total reflection, the neutron specular reflectivity profile can provide information about the scattering length density (SLD) profile perpendicular to the surface, and the layer thickness of the material. Typically, two graphs are here shown for each simulation/data set: (1) the reflectivity, R, as a function of the scattering vector for neutron momentum transfer, Q_z, where the data is shown with error bars, and a model fitted to the experimental data is shown as a solid, red curve, and (2) the fitted simulated neutron scattering length density as a function of depth position, z, where zero is defined at the interface between silicon and its native oxide. A schematic illustration of the model can be seen in **Figure S8**.

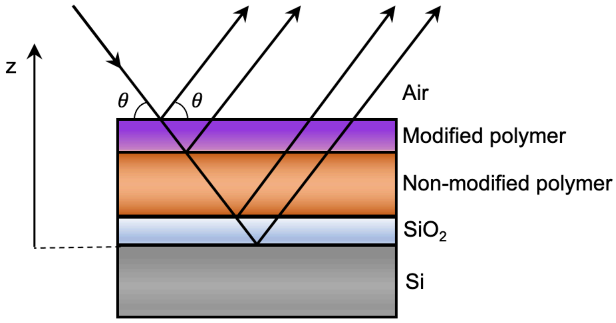

Figure S8. *Schematic illustration of neutron reflectometry and of the model used to interpret the data. Note that the incident neutron beam angle is significantly smaller than illustrated.*

Snell's law describes how a beam at incident angle θ_1 (to the interface) is refracted to an angle θ_2 by an interface of material with different refractive indices n_i according to

$$n_1 \cdot cos\theta_1 = n_2 \cdot cos\theta_2.$$

The refractive index n for neutrons can, by ignoring the absorption coefficient, be approximated as

$$n \approx 1 - \frac{\lambda^2}{2\pi} \cdot SLD.$$

Theoretical neutron scattering length densities (SLDs)

The theoretical neutron SLDs for different materials can be expressed as

$$SLD = N_A \cdot \rho \cdot \frac{\sum_i b_{c,i}}{\sum_i M_i},$$

where N_A is the Avogadro constant, ρ the density, b_c the bound coherent scattering length,[2,3] and M the molar mass of the substance, summarized for all constituent elements.[4] Calculated SLDs for relevant substances using this formula can be found in **Table S1**.

Table S1. *Calculated theoretical scattering length densities.*

**Material**	**Density / 10^3 (kg/m^3)**	**Sum of bound coherent scattering lengths / 10^{-15} m**	**Molar mass / 10^{-3} (kg/mol)**	**SLD / 10^{-6} Å^{-2}**
Si	2.33	4.15	28.1	2.07
SiO_2	2.65	15.8	60.1	4.18
Al_2O_3	3.95	24.3	102	5.67
MH ($C_{42}H_{72}O_{36}$)	1.85	219	1152	2.12
PS $(C_8H_8)_n$	1.00	23.3	104	1.35
H_2O	1.00	-1.68	18.0	-0.56
IPA (C_3H_8O)	0.786	-4.17	60.1	-0.33
Anisole (C_7H_8O)	0.995	22.4	108	1.24

Substrate

The model used for analysis of the neutron reflectivity data consisted of a silicon substrate (infinite thickness, SLD $2.07 \cdot 10^{-6}/Å^2$, roughness 2.65 Å), having a native oxide layer (5 Å thickness, SLD $4.16 \cdot 10^{-6}/Å^2$, roughness 1.56 Å) (see **Figure S9** and **Table S2**). This would correspond to an air inclusion of 0.5 vol% in the oxide layer, since the SLD of pure SiO_2 is $4.18 \cdot 10^{-6}/Å^2$.

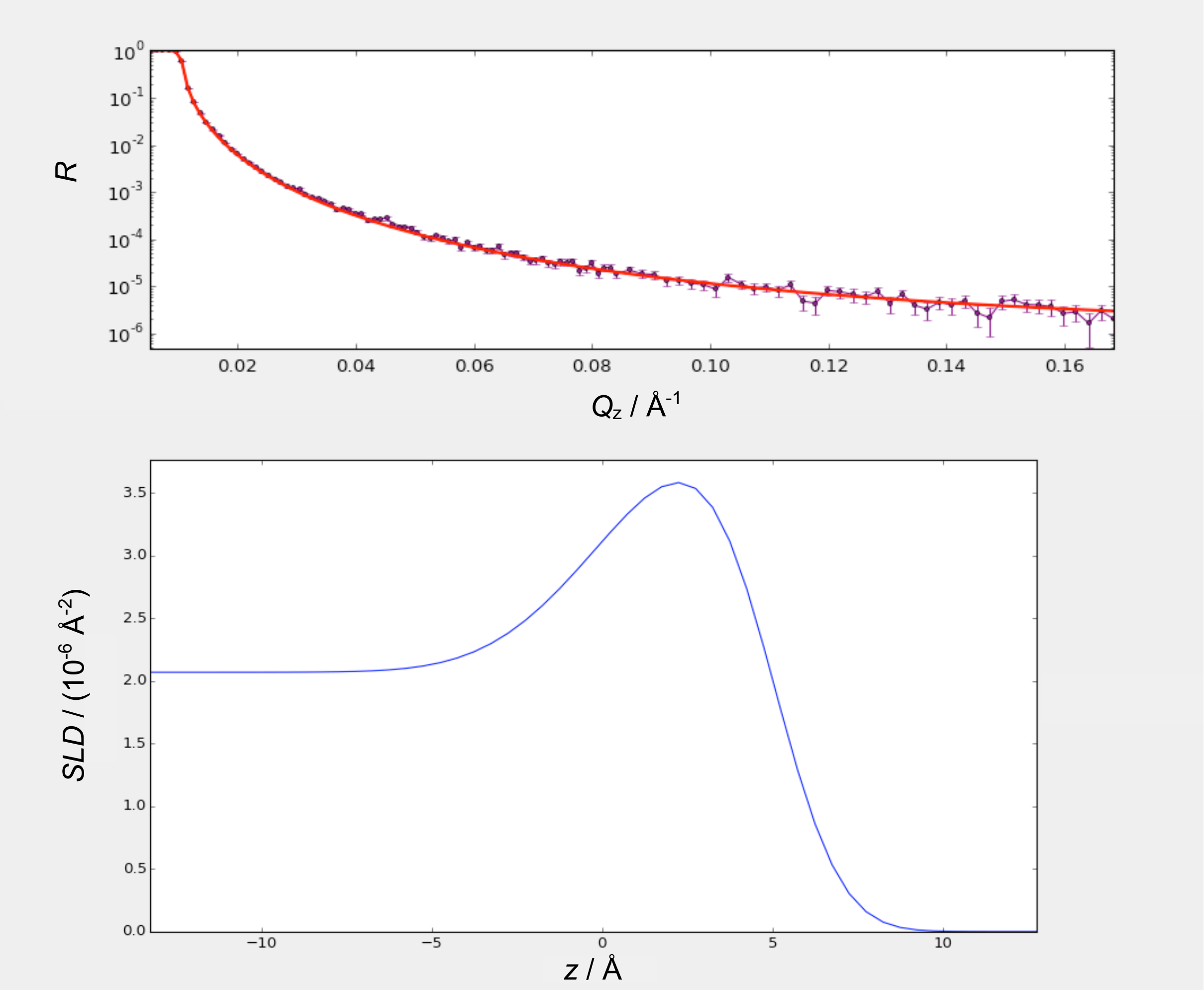

Figure S9. *NR data, simulated model and SLD for SiO_2 on Si.*

Table S2. *Neutron reflectivity analysis data from Si substrate.*

**Substrate**	
SiO_x thickness / Å	5
SiO_x SLD / 10^{-6} Å^{-2}	4.16
SiO_x top roughness / Å	1.6
Si SLD / 10^{-6} Å^{-2}	2.07
Si top roughness / Å	2.6

<u>Alumina</u>

An atomic layer deposition, using trimethyl aluminium and water, was made on top of a silicon substrate and measured in NR. The deposited layer thickness was measured to be 17.4 nm using ellipsometry. On top of the model for the silicon substrate, including the native oxide, one layer was added to represent alumina. NR data analysis of this alumina layer resulted in 171 Å thickness, SLD 4.49·10^{-6}/Å^2, roughness 5 Å (see **Figure S10**). This would correspond to a 21 vol% air inclusion into a pure Al_2O_3 layer (SLD 5.67·10^{-6}/Å^2). The NR analysis results are summarized in **Table S3**.

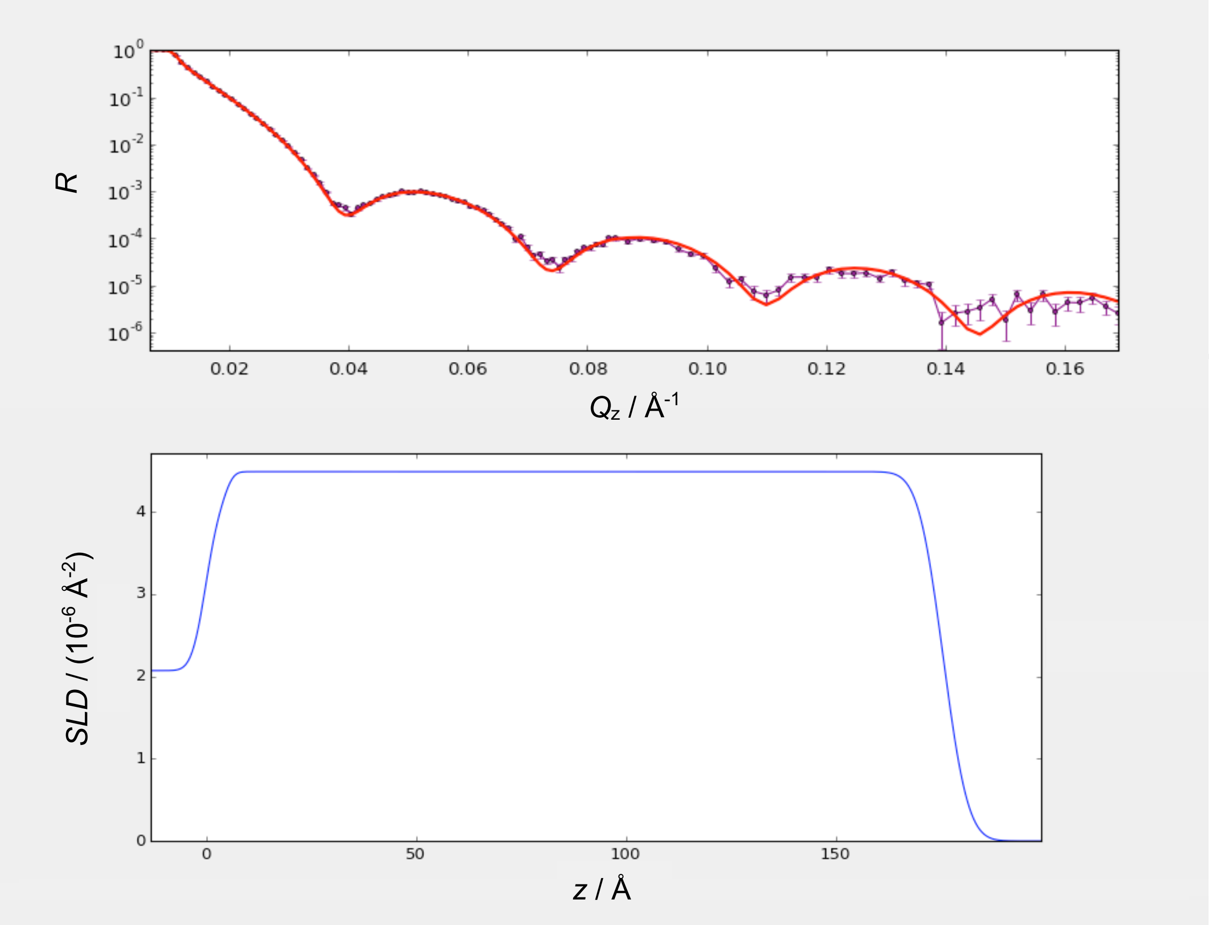

Figure S10. *NR data, simulated model and SLD for AlO_x on SiO_2 on Si.*

Table S3. *Neutron reflectivity analysis data from atomic layer deposited alumina.*

**ALD AlO_x**	
Thickness / Å	171
SLD / 10^{-6} Å^{-2}	4.49
Top roughness / Å	5
Included Al_2O_3 / vol%	79

PS-OH infiltration

Hydroxyl terminated polystyrene (PS-OH) was spin-coated upon a silicon substrate and measured in NR. The deposited layer thickness was measured to be 17.2 nm using ellipsometry. On top of the model for the silicon substrate, including the native oxide, one layer was added to represent PS-OH. NR data analysis of the PS-OH layer resulted in 178±2.5 Å thickness, SLD of (1.43±0.03)·10^{-6}/Å^2, roughness of 8 Å (see **Figure S11**).

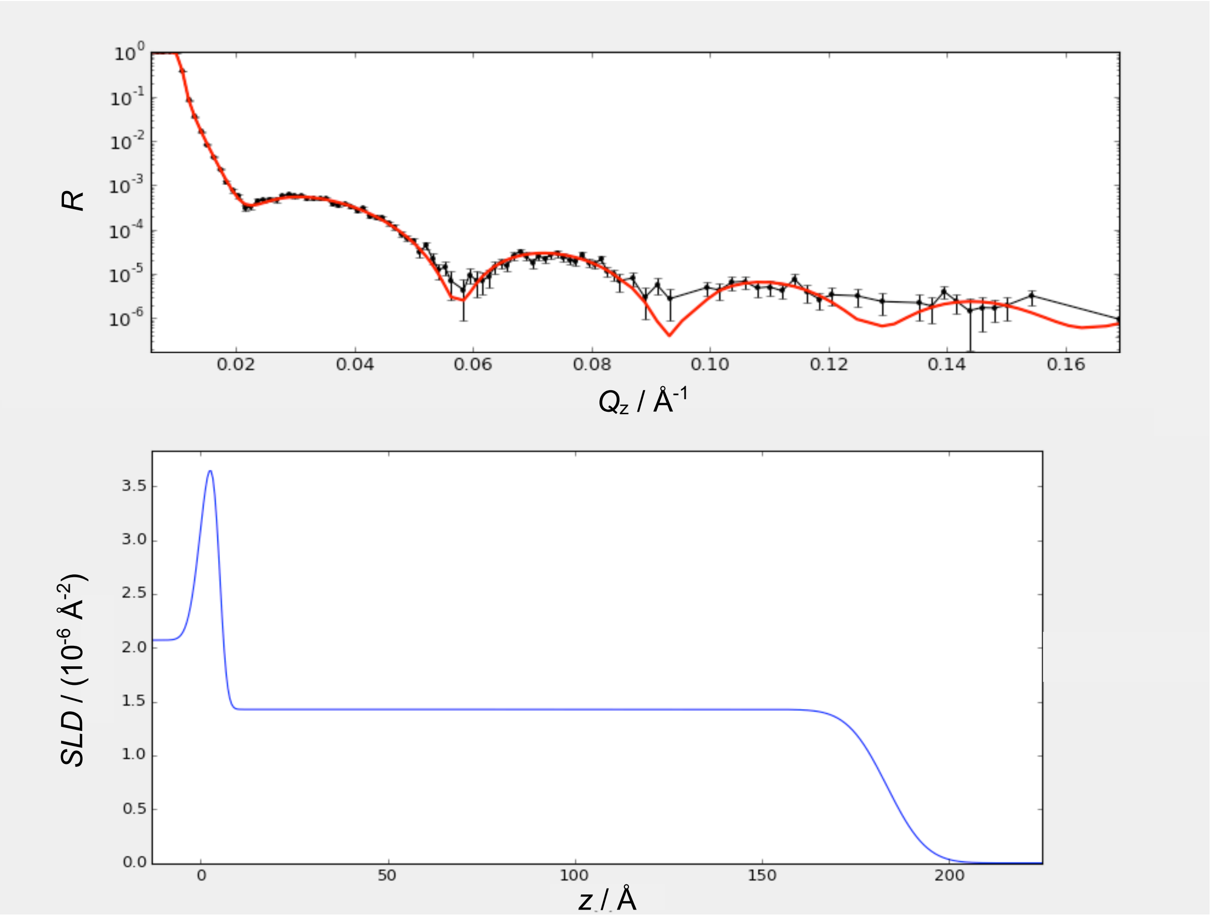

Figure S11. *NR data, simulated model and SLD for PS-OH on SiO_2 on Si.*

Dynamic PS-OH infiltration

After 2 dynamic SIS cycles into hydroxyl terminated polystyrene (PS-OH) on a silicon substrate, the sample was measured in NR. On top of the model for the silicon substrate, including the native oxide, one layer was added to represent the infiltrated PS-OH. NR data analysis of the 2-cycle infiltrated PS-OH layer resulted in 178±2.1 Å thickness, SLD of $(1.43\pm0.03)\cdot10^{-6}/\text{Å}^2$, roughness of 8 Å (see **Figure S12**). Thus, very similar SLD as the pristine PS-OH film.

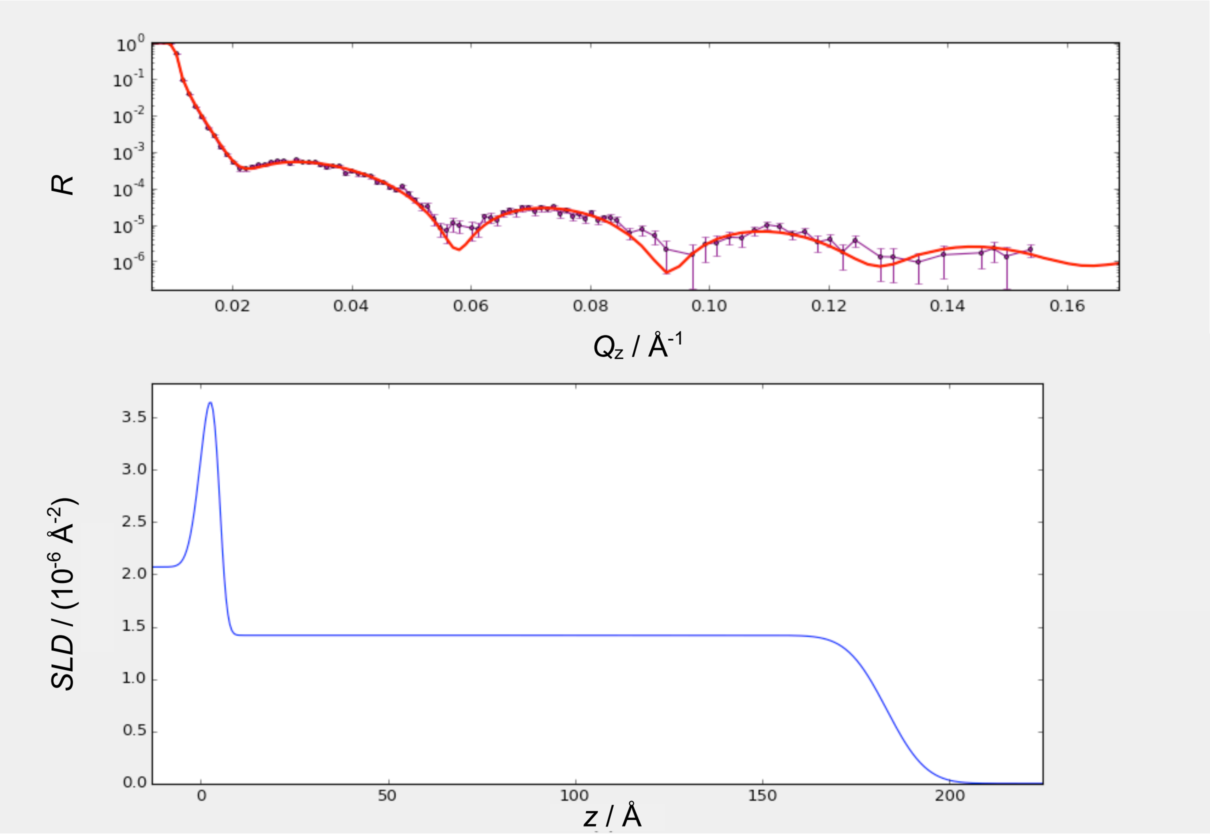

Figure S12. *NR data, simulated model and SLD for 2-cycle infiltrated PS-OH on SiO_2 on Si.*

After 8 dynamic SIS cycles into hydroxyl terminated polystyrene (PS-OH) on a silicon substrate, the sample was measured in NR. On top of the model for the silicon substrate, including the native oxide, one layer was added to represent the infiltrated PS-OH. NR data analysis of the 8-cycle infiltrated PS-OH layer resulted in 189±2.9 Å thickness, SLD of (1.46±0.04)·10^{-6}/Å^2, roughness of 8 Å (see **Figure S13**). The difference in layer thickness, comparing to the pristine PS-OH film, might be explained by variations from spin-coating, where 1 nm thickness difference is to be expected. There might be a slight addition of alumina in the hydroxyl terminated PS after 8 infiltration cycles. It would then be corresponding to an inclusion of 0.8 vol% pure Al_2O_3, or 1.1 vol% ALD Al_2O_3.

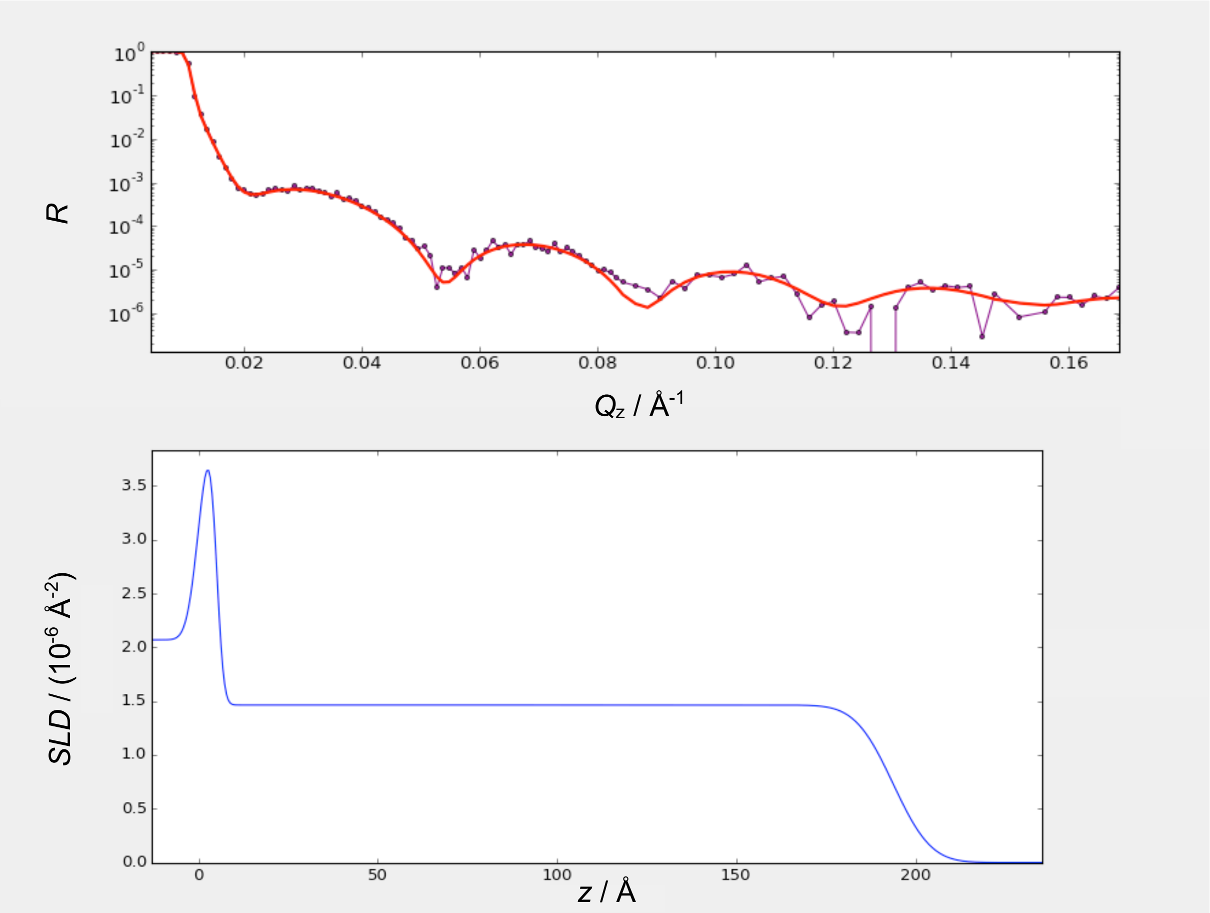

Figure S13. *NR data, simulated model and SLD for 8-cycle infiltrated PS-OH on SiO_2 on Si.*

Semi-static PS-OH infiltration

After 2 semi-static SIS cycles into hydroxyl terminated polystyrene (PS-OH) on a silicon substrate, the sample was measured in NR. On top of the model for the silicon substrate, including the native oxide, one layer was added to represent the infiltrated PS-OH. NR data analysis of the semi-statically infiltrated PS-OH layer resulted in 186±2.3 Å thickness, SLD of (1.42±0.03)·10^{-6}/Å^2, roughness of 8 Å (see **Figure S14**). Thus, very similar SLD as the pristine PS-OH film.

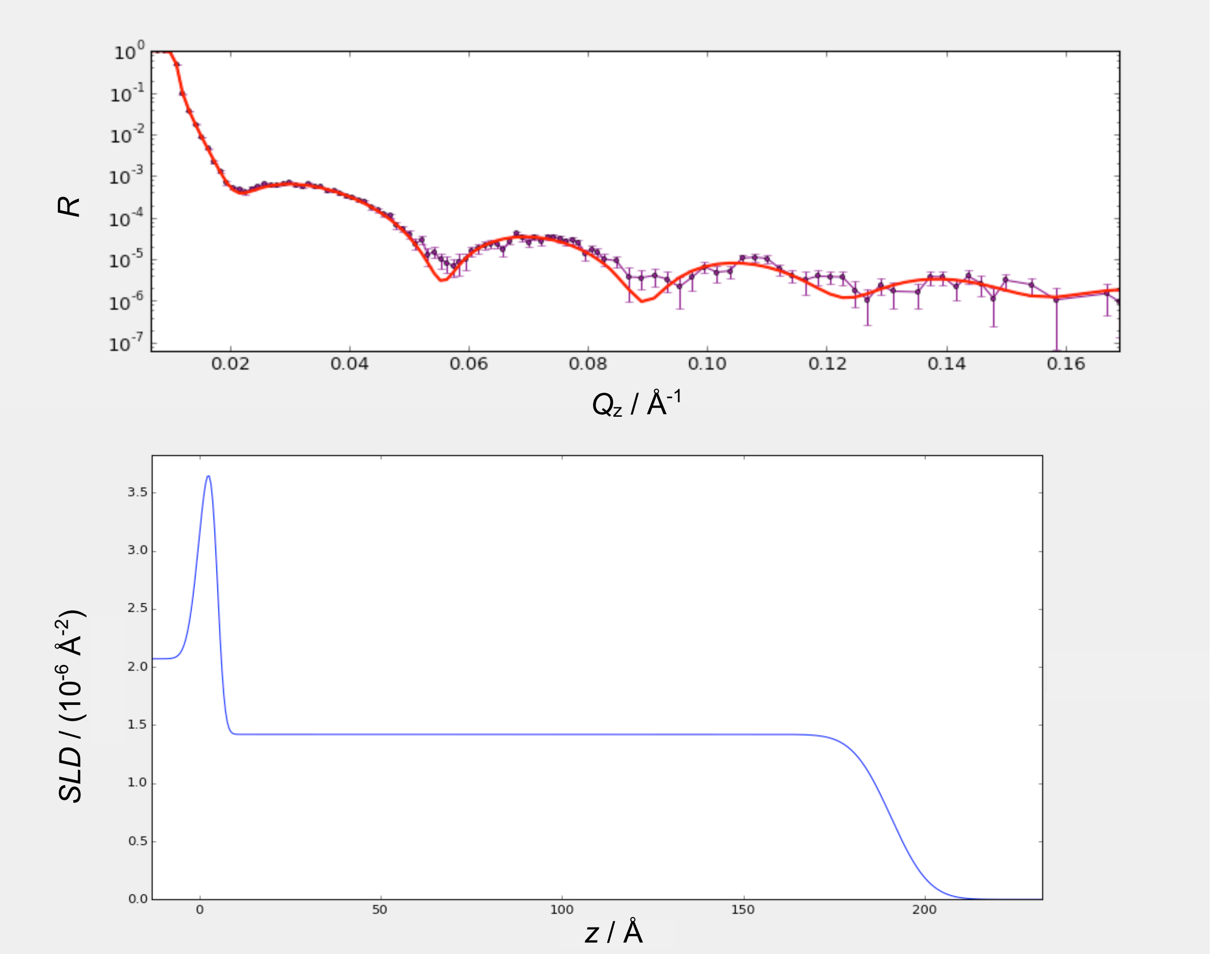

Figure S14. NR data, simulated model and SLD for 2-cycle semi-statically infiltrated PS-OH on SiO_2 on Si, with varying roughness.

Table S4. Neutron reflectivity analysis data from SIS of TMA and water into PS-OH.

PS-OH	Pristine	Dynamic				Semi-static
Number of infiltration cycles	0	1	2	4	8	2
Thickness / Å	178±3	-	178±3	-	189±3	186±3
SLD / 10^{-6} Å^{-2}	1.43±0.03	-	1.43±0.03	-	1.46±0.04	1.42±0.03
Top roughness / Å	8	-	8	-	8	8
Included Al_2O_3 / vol%	0	-	0	-	0.8	0

A summary of the NR analysis of TMA/H_2O infiltration into PS-OH can be seen in **Table S4**.

MH infiltration

Maltoheptaose (MH) was spin-coated upon a silicon substrate and measured in NR. The deposited layer thickness was measured to be 12.1 nm using ellipsometry. On top of the model for the silicon substrate, including the native oxide, one layer was added to represent the MH. NR data analysis of the MH layer resulted in 134±2.9 Å thickness, SLD of (1.63±0.04)·10^{-6}/Å^2, roughness of 5 Å (see **Figure S15**).

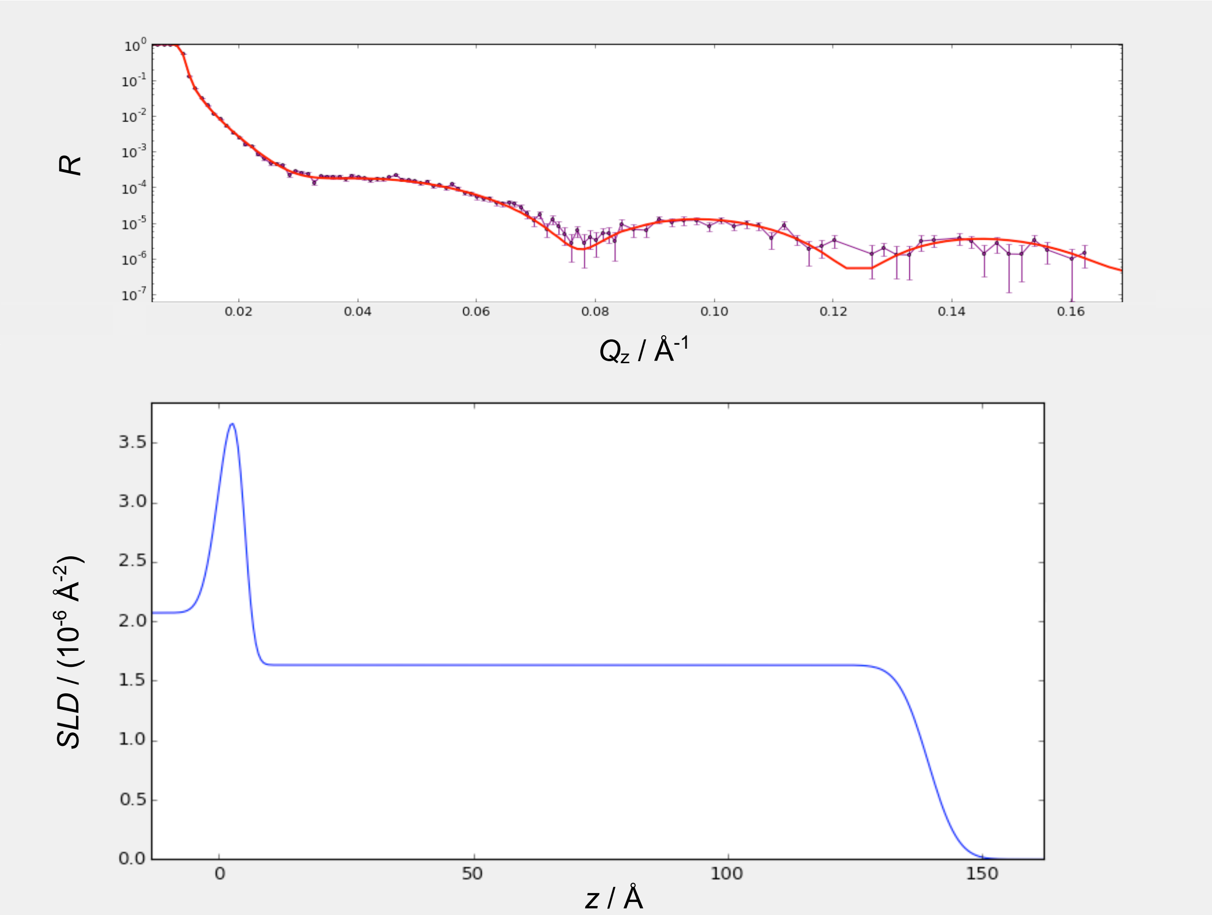

Figure S15. *NR data, simulated model and SLD for MH on SiO_2 on Si.*

When modelling the data for the infiltrated MH samples, a better fit was obtained when splitting the MH into two layers, where the top layer (having an interface to air) represents the infiltrated MH, and the lower layer (having an interface to the substrate) represents the unmodified MH. Regardless if a model with fix roughness of the lower layer and the top layer was used, or a model with varying roughness, the trend of increasing top layer SLD with number of cycles remained unchanged. For each infiltration cycle, the SLD increases, and after the 2nd cycle, the infiltration depth also increases. The model with varying roughness is, however, assumed to better describe the real situation.

Dynamic MH infiltration

After 1 dynamic SIS cycle into MH on a silicon substrate, the sample was measured in NR. On top of the model for the silicon substrate, including the native oxide, two layers were added, since a 1-layer model on top of the substrate was unsuccessfully fitted. The lower layer represents the unmodified MH, whereas the top layer represents the alumina enriched MH. NR data analysis of the 1-cycle infiltrated MH resulted in a top layer of 20±2.4 Å thickness, SLD of (1.80±0.12)·10^{-6}/Å^2, roughness of 5 Å, and underneath a layer of 118 Å thickness, SLD of 1.63·10^{-6}/Å^2, roughness of 19 Å (see **Figure S16**). This would correspond to 4 vol% pure Al_2O_3 mixed into our top MH layer, or to 6 vol% of ALD Al_2O_3.

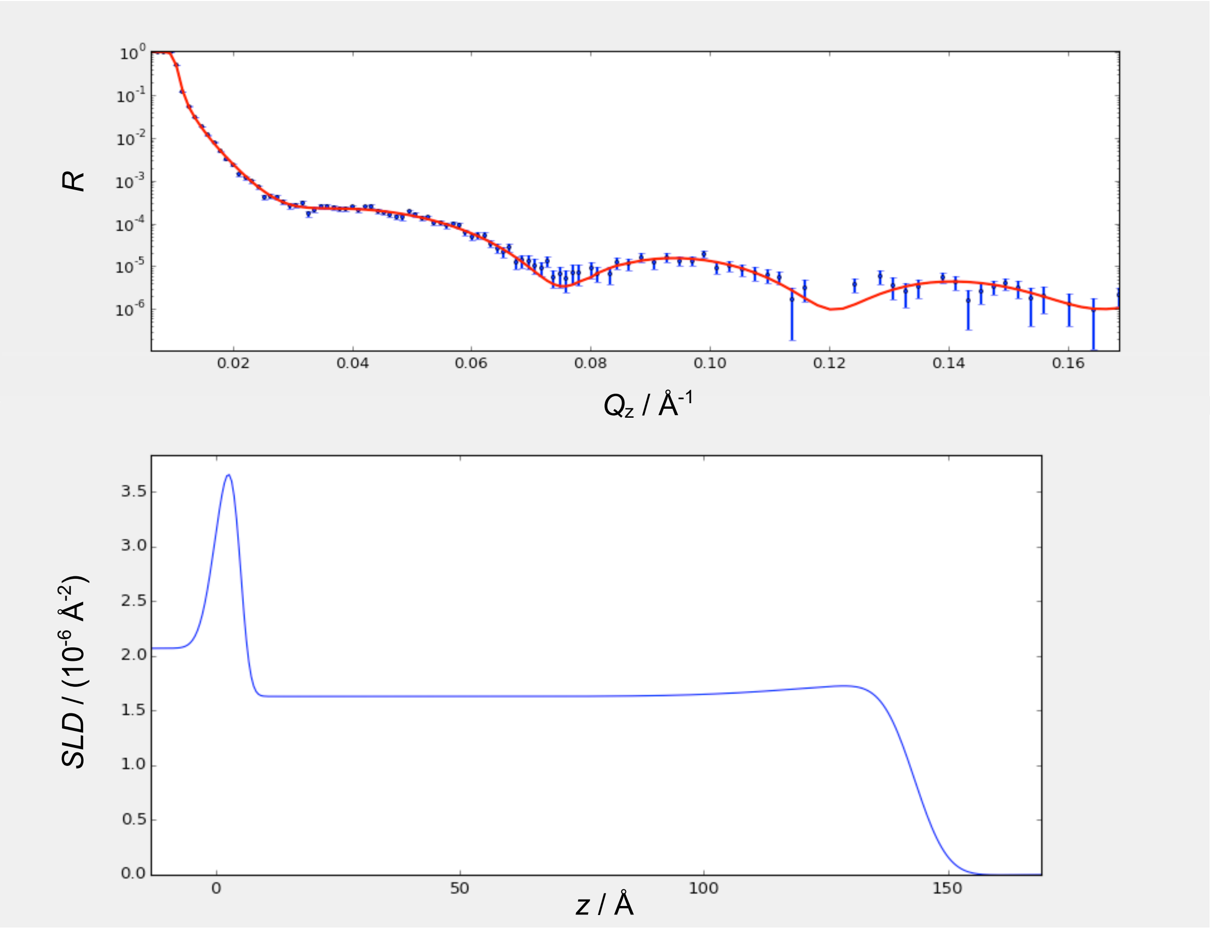

Figure S16. *NR data, simulated model and SLD for 1-cycle infiltrated MH on SiO_2 on Si, with varying roughness.*

After 2 dynamic SIS cycles into MH on a silicon substrate, the sample was measured in NR. On top of the model for the silicon substrate, including the native oxide, two layers were added, since a 1-layer model on top of the substrate was unsuccessfully fitted. The lower layer represents MH, whereas the top layer represents the alumina enriched MH. NR data analysis of the 2-cycle infiltrated MH resulted in a top layer of 19±2.5 Å thickness, SLD of $(2.05\pm0.16)\cdot10^{-6}/Å^2$, roughness of 8 Å, and underneath a layer of 119 Å thickness, SLD of $1.63\cdot10^{-6}/Å^2$, roughness of 9 Å (see **Figure S17**). This would correspond to 10 vol% pure Al_2O_3 mixed into the top MH layer, or to 14 vol% of ALD Al_2O_3.

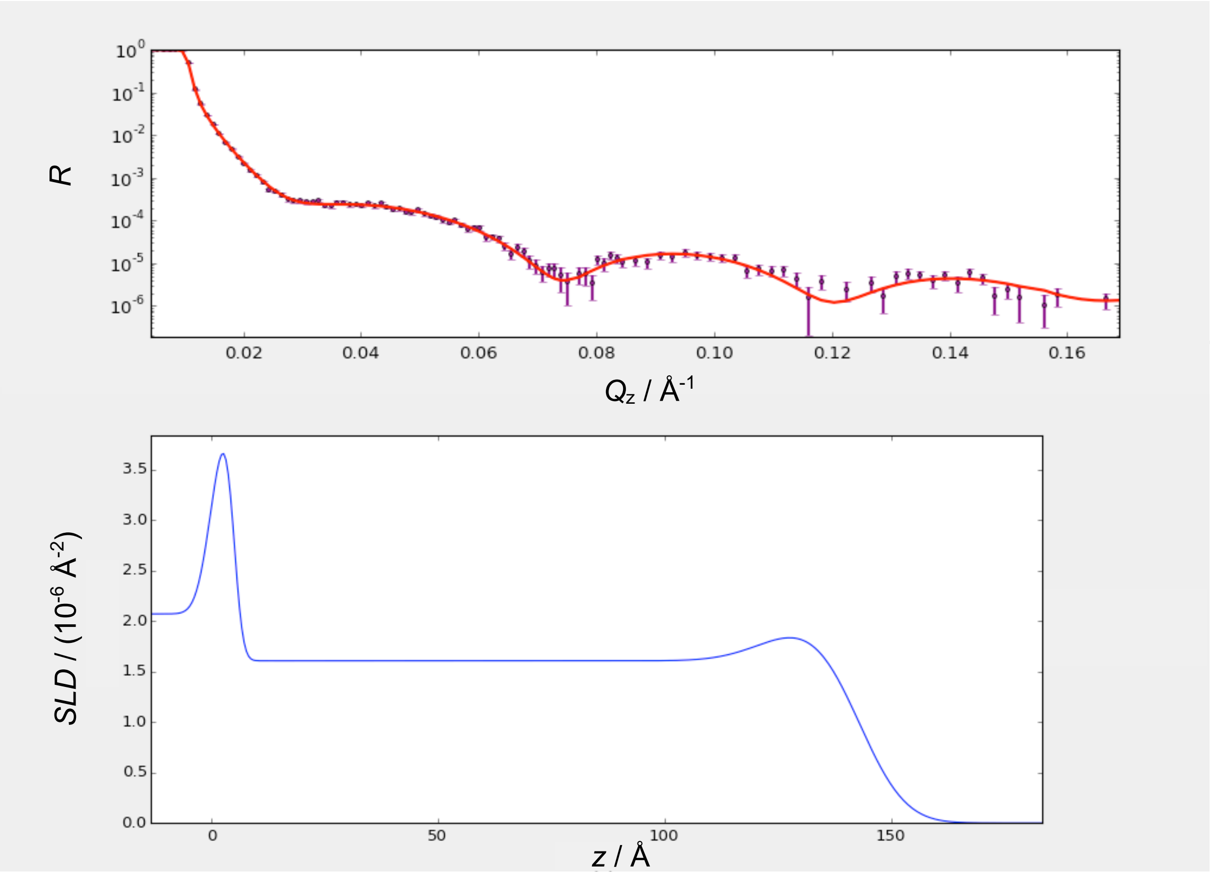

Figure S17. *NR data, simulated model and SLD for 2-cycle infiltrated MH on SiO_2 on Si, with varying roughness.*

After 4 dynamic SIS cycles into MH on a silicon substrate, the sample was measured in NR. On top of the model for the silicon substrate, including the native oxide, two layers were added, since a 1-layer model on top of the substrate was unsuccessfully fitted. The lower layer represents MH, whereas the top layer represents the alumina enriched MH. NR data analysis of the 4-cycle infiltrated MH resulted in a top layer of 22±2.9 Å thickness, SLD of (2.16±0.17)·10^{-6}/Å^2, roughness of 8 Å, and underneath a layer of 120 Å thickness, SLD of 1.63·10^{-6}/Å^2, roughness of 7 Å (see **Figure S18**). This would correspond to 13 vol% pure Al_2O_3 mixed into the top MH layer, or to 19 vol% of ALD Al_2O_3.

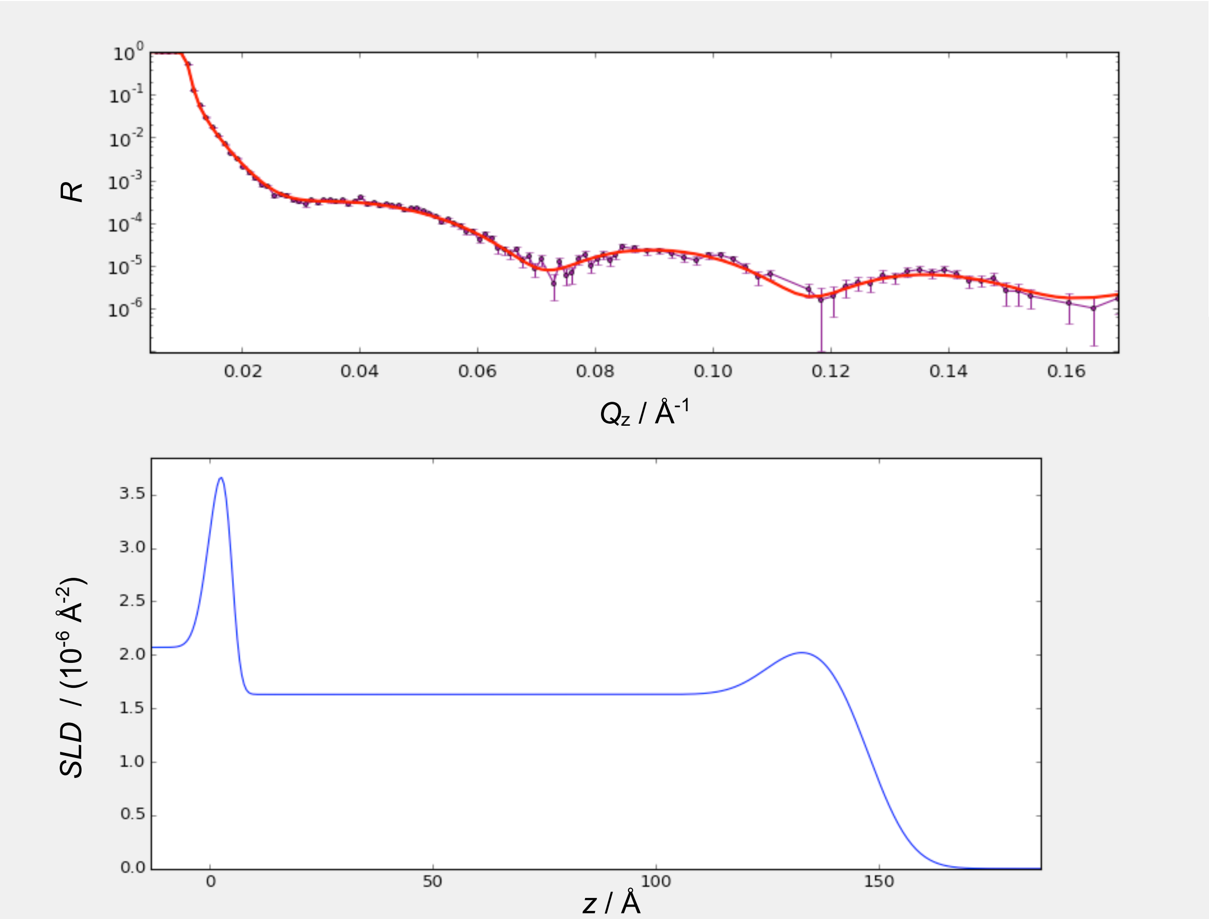

Figure S18. *NR data, simulated model and SLD for 4-cycle infiltrated MH on SiO_2 on Si, with varying roughness.*

After 8 dynamic SIS cycles into MH on a silicon substrate, the sample was measured in NR. On top of the model for the silicon substrate, including the native oxide, two layers were added, since a 1-layer model on top of the substrate was unsuccessfully fitted. The lower layer represents MH, whereas the top layer represents the alumina enriched MH. NR data analysis of the 8-cycle infiltrated MH resulted in a top layer of 25±2.6 Å thickness, SLD of $(2.54 \pm 0.17) \cdot 10^{-6}/\text{Å}^2$, roughness of 6 Å, and underneath a layer of 128 Å thickness, SLD of $1.63 \cdot 10^{-6}/\text{Å}^2$, roughness of 5 Å (see **Figure S19**). This would correspond to 23 vol% pure Al_2O_3 mixed into the top MH layer, or to 32 vol% of ALD Al_2O_3.

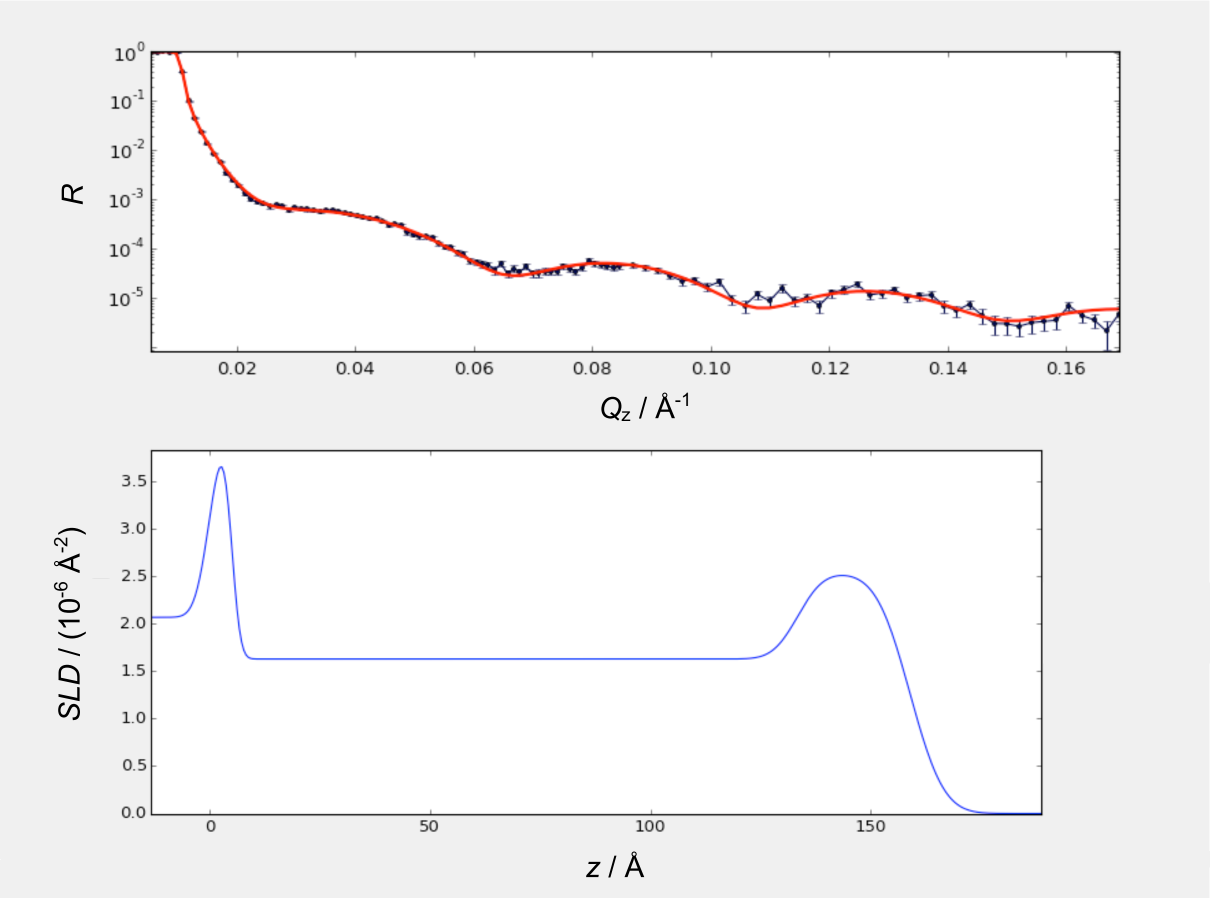

Figure S19. *NR data, simulated model and SLD for 8-cycle infiltrated MH on SiO_2 on Si, with varying roughness.*

Using the MH infiltration model with varying roughness of both lower layer and infiltrated layer, the SLD increases with number of infiltration cycles. After the first cycle, the infiltration depth is 20 Å, after 2 cycles, it is 19 Å. Thereafter, the infiltration depth increases with number of cycles.

Semi-static MH infiltration

After 2 semi-static SIS cycles into MH on a silicon substrate, the sample was measured in NR. On top of the model for the silicon substrate, including the native oxide, two layers were added, since a 1-layer model on top of the substrate was unsuccessfully fitted. The lower layer represents MH, whereas the top layer represents the alumina enriched MH. NR data analysis of the 2-cycle semi-statically infiltrated MH resulted in a top layer of 22±2.4 Å thickness, SLD of (1.92±0.16)·10^{-6}/Å^2, roughness of 8 Å, and underneath a layer of 118 Å thickness, SLD of 1.63·10^{-6}/Å^2, roughness of 11 Å (see **Figure S20**). This would correspond to 7 vol% pure Al_2O_3 mixed into the top MH layer, or to 10 vol% of ALD Al_2O_3.

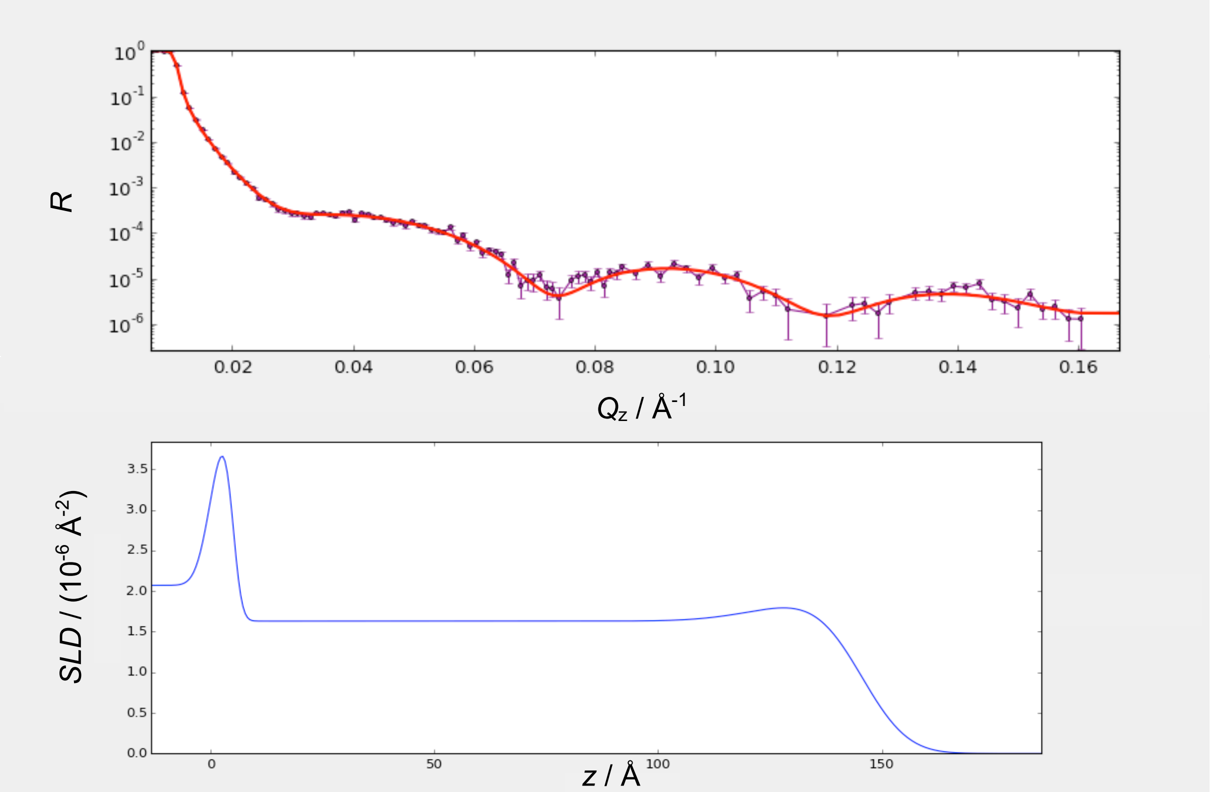

Figure S20. *NR data, simulated model and SLD for 2-cycle semi-statically infiltrated MH on SiO_2 on Si, with varying roughness.*

These results indicate that the semi-static infiltration depth after 2 cycles was 3 Å deeper than after 2 dynamic infiltration cycles, and equal to the depth after 4 dynamic infiltration cycles. However, the SLD after 2 semi-static cycles was 0.13·10^{-6}/Å^2 lower than after 2 dynamic cycles, and 0.12·10^{-6}/Å^2 higher than after 1 dynamic cycle.

Table S5. Neutron reflectivity analysis data from SIS of TMA and water into MH.

MH	Pristine		Dynamic		Semi-static
**Number of infiltration cycles**	**0**	**1**	**2**	**4**	**2**
Infiltration depth / Å	-	20±3	19±3	22±3	22±3
SLD modified layer/ 10^{-6} Å^{-2}	-	1.80±0.12	2.05±0.16	2.16±0.17	1.92±0.16
Top roughness modified layer / Å	-	5	8	8	8
Included Al_2O_3 / vol%	0	4	10	13	7
Unmodified layer thickness / Å	134±3	118	119	120	118
SLD unmodified layer / 10^{-6} Å^{-2}	1.63±0.04	1.63	1.63	1.63	1.63
Top roughness unmodified layer / Å	5	19	9	7	11

A summary of the NR analysis of TMA/H_2O infiltration into MH can be seen in **Table S5**.

Solubility

To evaluate solubility of trimethyl aluminium (TMA) and water precursors in PS and MH, tabulated values of the solubility parameter δ for PS of 18.7 (MPa)$^{1/2}$ and water of 48.0 (MPa)$^{1/2}$ were used, whereas estimations were made for MH and TMA using Hoy group contributions of molar attraction constants F (see **Table S6** and **Table S7**), and the expression

$$\delta = \frac{(\Sigma F)}{V} = \frac{(\Sigma F)\cdot\rho}{M_0},$$

where ρ is the density, and M_0 the molecular weight of the repeating unit[5,6]. The experimentally found density of 1.42 g/cm^3 for MH results in a solubility parameter of 26.3 (MPa)$^{1/2}$, whereas the tabulated density if 1.85 g/cm^3 gives 34.2 (MPa)$^{1/2}$. Using a density of 0.752 g/cm^3 for TMA, and ignoring any possible F contribution from Al, gives a solubility parameter of 9.5 (MPa)$^{1/2}$. These solubility estimations indicate that TMA should be more soluble in PS than in MH, whereas water should be more soluble in MH than in PS.

***Table S6**. Hoy group contributions to the total molar attraction constant for the repeating unit in MH.*

Group	F / (J cm^{-3})$^{1/2}$ mol^{-1}
2(-O-)	5·(235.3)
3(-OH)	3·(462.0)
>CH_2	269.0
5(>CH)	5·(176.0)
∑F	3005.6

Table S7. *Hoy group contributions to the total molar attraction constant for TMA.*

**Group**	**F / (J cm^{-3})$^{1/2}$ mol^{-1}**
3(-CH_3)	3·(303.4)
Al	N/A
∑F	≥910.2

N/A Not available.

References

1. I. Otsuka, K. Fuchise, S. Halila, S. Fort, K. Aissou, I. Pignot-Paintrand, Y. Chen, A. Narumi, T. Kakuchi and R. Borsali, *Langmuir*, 2010, **26**, 2325-2332.
2. V. F. Sears, *Neutron News*, 1992, **3**, 26-37.
3. Neutron scattering lengths and cross sections, https://www.ncnr.nist.gov/resources/n-lengths/, (accessed 27 January 2021).
4. T. P. Russell, *Materials Science Reports*, 1990, **5**, 171-271.
5. J. M. G. Cowie and V. Arrighi, *Polymers : chemistry and physics of modern materials*, CRC Press, 3. ed. edn., 2008.
6. A. F. M. Barton, *CRC Handbook of Solubility Parameters and Other Cohesion Parameters Handbook of Solubility Parameters and Other Cohesion Param*, CRC Press, 1983.
